# *m*-Terphenylamines, Acting as Selective COX-1 Inhibitors, Block Microglia Inflammatory Response and Exert Neuroprotective Activity

**DOI:** 10.3390/molecules28145374

**Published:** 2023-07-13

**Authors:** Damiano Rocchi, Juan F. González, Olmo Martín-Cámara, Maria Grazia Perrone, Morena Miciaccia, Antonio Scilimati, Celine Decouty-Pérez, Esther Parada, Javier Egea, J. Carlos Menéndez

**Affiliations:** 1 Unidad de Química Orgánica y Farmacéutica, Departamento de Química en Ciencias Farmacéuticas, Facultad de Farmacia, Universidad Complutense, 28040 Madrid, Spain; rocchid83@gmail.com (D.R.); jfgonzal@ucm.es (J.F.G.); olmomart@ucm.es (O.M.-C.); 2 Dipartimento di Farmacia—Scienze del Farmaco, Università degli Studi di Bari Aldo Moro, 70121 Bari, Italy; mariagrazia.perrone@uniba.it (M.G.P.); morena.miciaccia@uniba.it (M.M.); antonio.scilimati@uniba.it (A.S.); 3 Molecular Neuroinflammation and Neuronal Plasticity Research Laboratory, Hospital Universitario Santa Cristina, Instituto de Investigación Sanitaria-Hospital Universitario de la Princesa, 28009 Madrid, Spain; celinedecouty96@gmail.com (C.D.-P.); esther.parada@educa.madrid.org (E.P.)

**Keywords:** terphenylamines, COX-1 inhibitors, neuroinflammation, oxidative stress, neuroprotection

## Abstract

Inhibition of cyclooxygenase-2 (COX-2) has been extensively studied as an approach to reduce proinflammatory markers in acute brain diseases, but the anti-neuroinflammatory role of cyclooxygenase-1 (COX-1) inhibition has been rather neglected. We report that *m*-terphenylamine derivatives are selective COX-1 inhibitors, able to block microglia inflammatory response and elicit a neuroprotective effect. These compounds were synthesized via a three-component reaction of chalcones, β-ketoesters, and primary amines, followed by hydrolysis/decarboxylation of the ester group. Together with their synthetic intermediates and some urea derivatives, they were studied as inhibitors of COX-1 and COX-2. The *m*-terphenylamine derivatives, which were selective COX-1 inhibitors, were also analyzed for their ability to block microglia inflammatory and oxidative response. Compound **3b** presented an interesting anti-inflammatory and neuroprotective profile by reducing nitrite release, ROS overproduction, and cell death in organotypic hippocampal cultures subjected to LPS. We thus show that COX-1 inhibition is a promising approach to provide enhanced neuroprotection against acute inflammatory processes, which are crucial in the development of a plethora of acute neurodegenerative injuries.

## 1. Introduction

Cyclooxygenase (COX) is the main enzyme responsible for the conversion of arachidonic acid (AA) into prostaglandins (PGs) and reactive oxygen species (ROS), both of which participate actively in the neuroinflammatory process. There are two different isoforms of COX, namely COX-1 and COX-2, that differ mainly in tissue expression, mechanism of regulation, and the nature of the upstream and downstream-coupled enzymes. COX-1 is the isoform constitutively expressed and is responsible for the constitutive synthesis of PGs [1]. On the other hand, COX-2 is induced in response to inflammatory stimuli, and for this reason, selective inhibition of COX-2 can reduce inflammation without affecting the physiological functions of COX-1-derived PGs.

In recent years COX-1 has attracted much attention because it is constitutively expressed in various areas of the brain, with a predominant localization in microglia, where it has a proinflammatory role in the pathophysiology of neurological disorders [2]. In fact, COX-1 knockout mice are more resistant than their wild-type littermates to LPS-induced neuronal death and show a reduction in the inflammatory response [3], an effect that has also been noticed with COX-1 selective inhibitors [4]. In contrast, the neuroinflammatory response is exacerbated in LPS-treated COX-2 knockout mice. These results have been explained by the observation that COX-1 catalyzes the transformation of AA into the proinflammatory PGE_2_, while in the same conditions, COX-2 catalyzes the conversion of AA into its protective lipid metabolites. These observations suggest that selective COX-1 inhibitors could be useful pharmacological tools to reduce brain inflammation.

Neuroinflammation is one of the pathological hallmarks of many CNS disorders, such as Alzheimer’s and Parkinson’s disease, as well as cerebral ischemia [5,6,7,8]. In the CNS, microglia cells (the macrophages of the brain) play a crucial role in immune response and tissue repair [9]. Microglia activation in response to cerebral insults produces an acute release of proinflammatory mediators such as nitric oxide (NO), inflammatory cytokines, prostaglandin E_2_ (PGE_2_), and reactive oxygen species, among others [10,11,12]. In the course of these diseases, the dysfunction of microglial cells enhances their neurotoxic effects and removes their neuroprotective functions, thereby contributing to chronic inflammation. For this reason, inhibition of COX-1 can be viewed as a promising therapeutic mechanism in the treatment of neuroinflammatory diseases. Thus, two different COX-1 inhibitors (valeryl salicylate and SC-560) reduce hippocampal PGE_2_ production subsequent to cerebral ischemia, showing that COX-1 is dramatically involved in neuronal death of hippocampal neurons [13,14]. Interestingly, it has been shown that COX-1 knockout mice are more vulnerable to focal cerebral ischemia [14]. Moreover, the progression of Alzheimer’s disease has also been related to the expression of COX-1, and in vivo imaging experiments of COX-1 have found a strong expression of COX-1 surrounding amyloid plaques [15]. Based on these findings, a new class of compounds with dual activity by inhibiting both COX-1 as well as amyloid aggregation is under study as promising agents in the treatment of amyloid diseases [16]. COX-1 has also been shown to mediate neuroinflammation and neurotoxicity in a mouse model of retinitis pigmentosa [17]. In summary, the pharmacological modulation of microglial activation via selective COX-1 inhibition [18] is an attractive, if little explored, approach to therapy [2] and diagnosis [19] in CNS inflammatory diseases, and also to other inflammation-connected diseases, including cancer [20]. In the context of the therapeutic use of COX-1 inhibitors, it must be stressed that, in spite of previous misconceptions, animal studies suggest that the total extent of COX inhibition is more important for gastric toxicity than the extent of the inhibition of the individual COX-1 and COX-2 enzymes and for this reason highly selective COX-1 inhibitors do not exhibit particularly high gastrointestinal toxicity, behaving similarly to highly selective COX-2 inhibitors [21,22,23].

There are few known selective COX-1 inhibitors, and they are usually classified according to their chemical structures into five families, namely diarylheterocycles, benzamides and nicotinamides, arylpropionic acids, stilbenes and miscellaneous inhibitors [24,25]. We describe here the discovery of a new family of compounds that hold promise in this area, namely *m*-terphenylamines, together with their characterization as neuroprotective agents. *m*-Terphenyl derivatives have shown promise in several therapeutic areas. For instance, 19DAP02, an *m*-terphenyl-based diamidine, and its isosteres seem good preclinical candidates for the treatment of second-stage African sleeping sickness [26], and a m-terphenyl carbamate (LUF5771) was the first small-molecule allosteric inhibitor of the luteinizing hormone receptor [27]. The *m*-terphenylamine framework bears similarity to other structures known to behave as COX-1 inhibitors, such as diarylheterocycles. A few *m*-terphenylamines have been reported to be non-selective COX inhibitors, although the enzyme inhibition measurements were performed only at two concentrations, i.e., 10 µM and 100 µM [28]. Reasoning that IC_50_ data are necessary for the proper characterization of these compounds and that this study might perhaps lead to uncovering additional interesting properties, we set out to synthesize and study a representative library of derivatives of the *m*-terphenylamine framework.

## 2. Results and Discussion

### 2.1. Synthesis

**Scheme 1:**
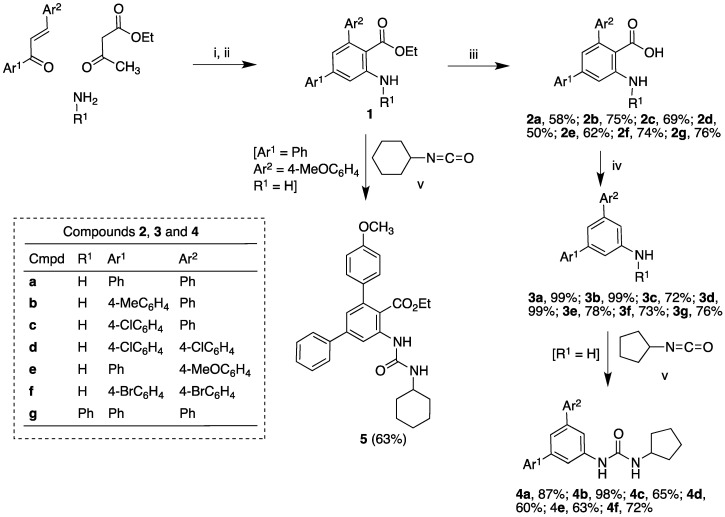
Synthesis of compounds **2**–**5**. Reagents and conditions: i. For **2a**–**f**: butylamine, CAN (5%), ethanol, microwave (150 °C, 200 W), 2.5 h; then, ammonium formate, microwave (150 °C, 200 W), 1 h. For **2g**: aniline, CAN (5%), ethanol, reflux, 72 h [30]; ii. DDQ, toluene, rt, 2 h. iii. NaOH, EtOH, reflux, 24 h; iv. NaOH, DMF, MW, 180 °C, 90 min; v. CH_2_Cl_2_, rt, 18 h.

The synthetic route is summarized in Scheme 1 and starts with the preparation of the *m*-terphenyl-embedded anthranilic ester derivatives in **1** by application of our previously described three-component reaction of chalcones, β-ketoesters, and primary amines, followed by dehydrogenation [29,30]. Basic hydrolysis of the ester group afforded anthranilic acids in **2**, which were decarboxylated to terphenylamines in **3** under microwave irradiation at 180 °C in the presence of sodium hydroxide in dimethylformamide solution. Since compounds containing urea motifs are widely employed in drug design due to their hydrogen-binding ability [31] and bearing in mind the presence of an arginine residue at the COX active site, we also planned the synthesis of derivatives in **4**, containing a urea moiety, which can be expected to interact with this residue by hydrogen bonding [32]. Finally, for comparison purposes, we also prepared an additional urea derivative in **5** from one of the anthranilic esters.

### 2.2. COX Inhibition Studies

**Figure 1:**
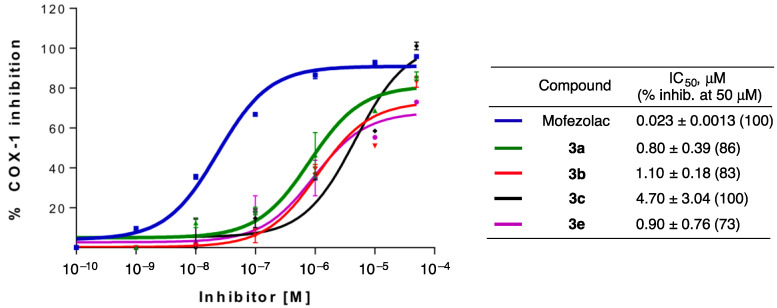
Concentration-response curves and IC_50_ values for the most promising compounds **3a**–**e** and mofezolac, used as reference compounds. Experiments were performed in triplicate.

Compounds **1**–**5** were screened for their ability to inhibit ovine COX-1 and human COX-2, using as positive controls the COX-1 inhibitor mofezolac and the COX-2 inhibitor celecoxib. All compounds were studied at a single dose of 50 µM, and the results of this study are shown in Table 1 and Figure 1, and the main conclusions obtained are:

(a) Amino esters in **1** lack significant activity, although some of them (**1c** and **1d**) slightly inhibited COX-1 at 50 μM.
(b) The corresponding amino acids in **2** do not achieve significant inhibition levels either, although they are slightly more active. They seem to be selective towards COX-1.
(c) Amino-*m*-terphenyls in **3** were the most interesting derivatives, and most of them showed COX-1 inhibition at IC_50_ values in the μM range. Moreover, three of these compounds (**3b**, **3c**, and **3e**) showed a complete selectivity towards COX-1, and a fourth (**3a**) showed a COX-1/COX-2 inhibition ratio around 7. COX-1 inhibition and COX-1/COX-2 selectivity are not connected with the electron density of the aromatic rings since similar results have been obtained for compounds bearing no substitution (**3a**), electron-withdrawing groups (**3c**, **3d**) and electron-releasing groups (**3e**). This level of selectivity had not been previously observed for this type of compound and is similar to or better than the one found in other families of COX-1 inhibitors currently being developed, such as diarylisoxazoles [33].
(d) Urea derivatives in **4** and **5** are essentially inactive. The only example with some activity (compound **4f**) did not show COX-1 selectivity.

The binding of compounds **3** to COX-1 was studied computationally, using as the basis the crystallographic structure of the mofezolac-COX-1 complex (PDBid 5WBE) [34]. For comparison purposes, we also performed docking calculations with celecoxib. We obtained a binding mode similar to that of mofezolac but with no hydrogen bonding and a less favorable binding energy, which was expected since celecoxib is a selective COX-2 inhibitor. Since the binding region in COX-1 is small and irregular, it is difficult for rigid and large molecules to fit into it. Docking studies revealed that, among our molecules, only those belonging to family **3** are small enough to fit inside this binding pocket, and among them, only the ones bearing no substituent or only one substituent in any aryl ring and a primary amino group (**3a**, **3b**, **3c** and **3e**) fitted the active site properly (Table 2).

**Figure 2:**
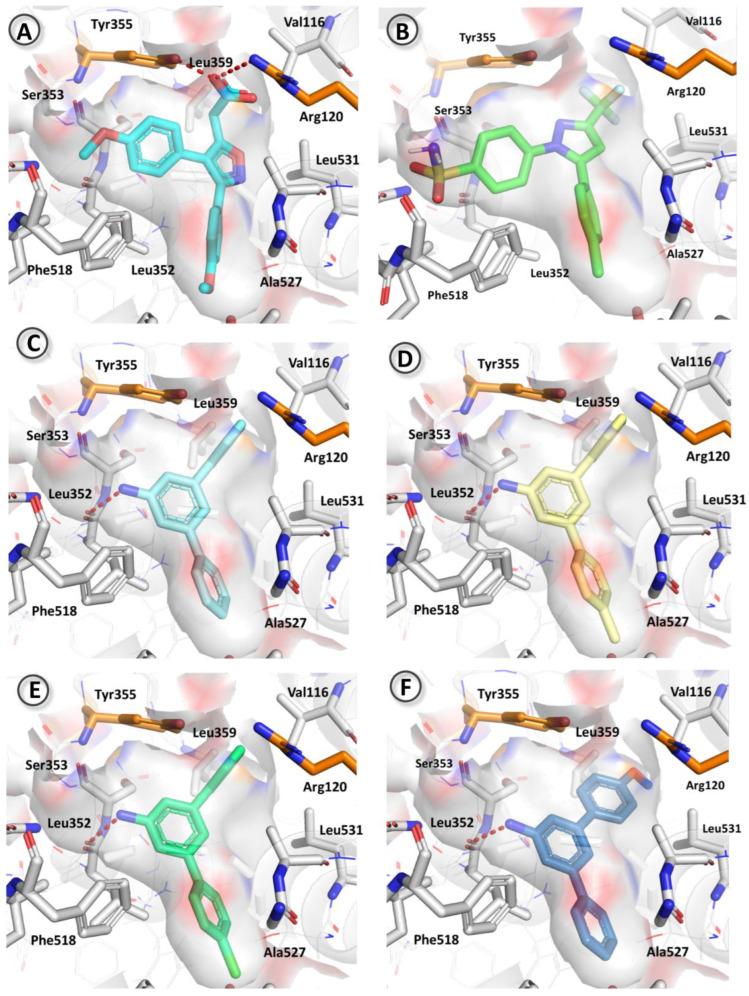
Binding pose of mofezolac (**A**), celecoxib (**B**), and most favorable binding poses for compounds **3a** (**C**), **3b** (**D**), **3c** (**E**), and **3e** (**F**). The binding pocket is represented by the white surface, and the residues that interact with ligands are displayed as white sticks, except for the residues at the entrance of the binding pocket, Arg120 and Tyr355, which are displayed in orange.

These four compounds shared a common binding mode, which mainly involves a hydrogen bond between the primary amine (donor) and the backbone oxygen from Leu352, together with some hydrophobic interactions with Val116, Val349, Leu352, Tyr355, Leu359, Phe518, Gly526, and Ala527. As can be observed in Figure 2, ligands **3b**, **3c**, and **3d** fitted tightly to the restricted space in this pocket, and therefore any additional substitution would hinder fitting. This particular binding mode shared most hydrophobic interactions with the binding pose corresponding to the reference compound mofezolac, except for some important interactions between the *p*-methoxyphenyl placed near the catalytic Tyr385. On the other hand, polar contacts are completely different since mofezolac lacks the hydrogen bond with Leu352 observed for the terphenylamine derivatives but did show a salt bridge with Arg120 and three hydrogen bonds, two with Arg120 and one with Tyr355, resulting in a much stronger interaction [35]. These differences explain the difference in potency exerted by compounds **3** and mofezolac.

### 2.3. Blockade of Microglia Inflammatory and Oxidative Responses

The COX-1 inhibition data prompted us to undertake further work on the activity of compounds **3** in a neuroinflammation situation. Once we had verified that the compounds studied were not toxic per se in microglia BV2 cells (see the data in Table S1), we measured the nitrites released to determine the efficacy of our compounds in reducing microglia activation. Nitric oxide is a potent mediator of the classic activated microglia phenotype (M1); in fact, the lack of nitrite release correlated with an anti-inflammatory microglia (M2) phenotype [36]. Therefore, we evaluated the response of the BV2 microglia cells to a challenge of 1 μg/mL LPS. For this purpose, BV2 microglia cells were incubated with LPS, a well-known agonist of the toll-like receptor 4 (TLR4) that causes a neuroinflammatory response, in combination with increasing concentrations of our COX-1 inhibitors (1, 3, and 10 μM). Nitrite release was measured at 24 h and 48 h post-LPS-insult, and the highly selective COX-1 inhibitor SC-560 was used as a positive control [37]. As shown in Table 3, compounds **3a**, **3b**, and **3e** exhibited comparable nitrite reduction after 24 h of LPS incubation. However, compound **3c**, less potent as a COX-1 inhibitor, required higher concentrations to achieve maximum anti-inflammatory response. Similar results were observed after 48 h of the LPS treatment. Importantly, the positive control SC-560 (100 nM), a selective COX-1 inhibitor, also exhibited a significant effect in reducing nitrites after 24 and 48 h of LPS toxic stimuli.

**Figure 3:**
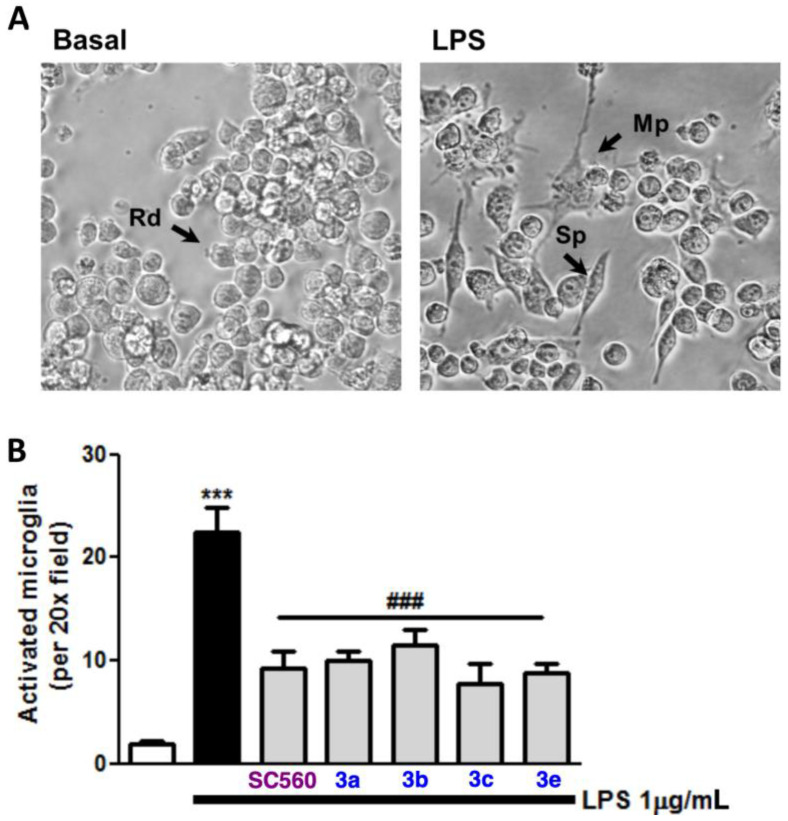
Effect of COX-1 inhibitors **3** on LPS-induced microglia activated phenotype. Panel (**A**) shows microphotographs (original magnification 20×) of non-treated BV2 microglia and LPS-treated cells. Panel (**B**) shows statistics of the effect of COX-1 inhibitors on activated phenotype elicited by LPS treatment. Arrows indicate the phenotype analyzed: spindle (Sp), multipolar (Mp), and rounded (Rd). Data are expressed as means ± SEM of at least four different cell batches in triplicate. *** *p* < 0.001 with respect to basal cells; ### *p* < 0.001 in comparison with LPS-treated cells.

Next, we set out to study the changes in microglia phenotype under this toxicity protocol. As mentioned above, microglia are comprised of variable morphology cells that are always adapting to the surrounding media and, therefore, to physiopathological conditions. Indeed, morphological appearance is well correlated with the different states of microglia activation [38,39]. The microglia-activated phenotype (M1), which is the classical state of this kind of cell in response to proinflammatory insults such as LPS, is linked with the overexpression of a plethora of cytokines and inflammatory markers, namely TNF, IL-1β, IL-6, MCP-1 or CD11b [40,41,42]. All of these markers are commonly associated with morphological changes towards an amoeboid phenotype in primary cultures or in in vivo studies. Nevertheless, it has been recently described that BV2 microglia, as well as macrophages, turn from rounded to spindle-shaped or multipolar phenotypes in response to various M1 proinflammatory stimuli such as LPS/IFNγ or spinal cord injury conditioned media [43,44]. In this context, we assessed the ability of our compounds to prevent the morphological changes induced by LPS by analyzing the percentage of BV2 ramified cells (multipolar or spindle) over the total BV2 per field following cell incubation with our compounds and 1 μg/mL LPS for 24 h. As shown in Figure 3, compounds **3a**, **3b**, and **3e** (at 3 μM) and **3c** (at 10 μM), as well as the positive control, reduced the number of BV2 ramified cells by 60%. The protective effect associated with COX-1 inhibition is in agreement and complements the discovery that selective COX-1 inhibitors reduced the phosphorylation of IkB and PGE_2_ levels induced by LPS in N13 microglial cells [45].

**Figure 4:**
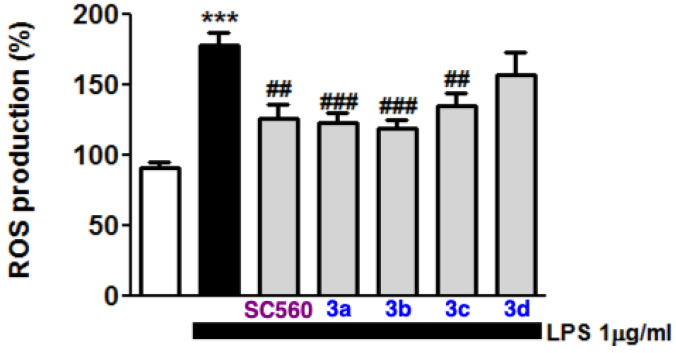
Effect of COX-1 inhibitors **3** on LPS induced microglia ROS production. Statistics of the effect of COX-1 inhibitors on ROS production induced by LPS treatment are shown. Data are expressed as means ± SEM of at least four different cell batches in triplicate. *** *p* < 0.001 with respect to basal cells; ### *p* < 0.001, ## *p* < 0.01 in comparison with LPS-treated cells.

Activation of toll-like receptors (TLRs), particularly the TLR4 subtype, is closely associated with the production of reactive oxygen and nitrogen species that contribute to neuronal death in the CNS. In this connection, NADPH oxidase enzymes are the main ones responsible for ROS production induced by NF-κB nuclear translocation [46]. For this reason, we decided to assess the ROS levels of BV2 microglia cells treated with LPS and the effect of our compounds under this toxicity protocol. To this end, the compounds, at different concentrations (3 μM of **3a**, **3b**, and **3d** and 10 μM of **3c**) were incubated with 1 μg/mL LPS for 24 h. After this period, ROS production was measured with the fluorescent probe H2DCFDA. Treatment with LPS increased by 1.75-fold the amount of ROS compared with control cells. Compounds **3a**, **3b**, and **3c**, at 3 μM, as well as the positive control SC-560 at 100 nM, significantly reverted ROS production almost to basal levels. However, compound **3d** failed to decrease the levels of ROS in BV2 microglia cells subjected to LPS (Figure 4).

### 2.4. Compound 3b Protects against LPS Challenge in Organotypic Hippocampal Slices

**Figure 5:**
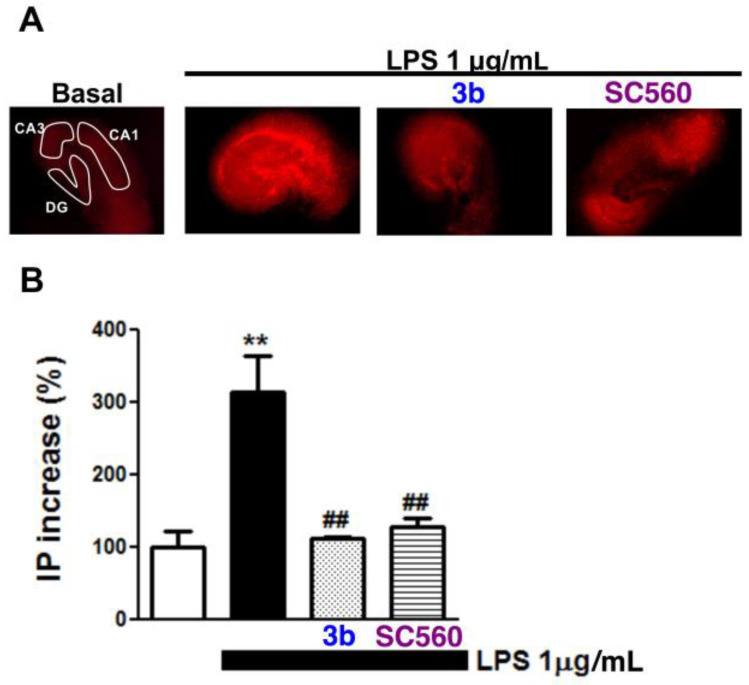
Protective effect of COX-1 inhibitor **3b** in organotypic hippocampal cultures (OHCs) treated with LPS. Panel (**A**) shows microphotographs (original magnification 2×) of the hippocampal subfields loaded with PI. Slices were treated with LPS-1 µg/mL for 24 h in combination with positive control SC-560 or **3b**. Panel (**B**) shows statistics of PI increase in the CA1 subfield of the OHCs. Values are expressed as mean ± SEM of at least four independent experiments; ** *p* < 0.01 with respect to the basal; ## *p* < 0.01 in comparison with LPS-treated slices.

With these promising data in hand, we selected compound **3b** for its study in a more complex preparation. In vivo administration of LPS produces a proinflammatory cascade that causes memory deficits, an increase of inflammatory markers, and impairment of hippocampal glutamatergic transmission [47,48,49,50], contributing to neuronal death [51]. All of these functional changes have been described to take place mainly in the hippocampus region. Thus, we subjected organotypic hippocampal cultures (OHCs) to LPS (1 μg/mL) to assess the protective effect of COX-1 inhibition. This kind of explant allows us to study the microglia-neuron network in a more complex and physiological model of cerebral insult [52]. The hippocampal slices incubated with LPS for 24 h exhibited a marked propidium iodide (PI) uptake in the CA1 hippocampus field in comparison to control slices, which was prevented by treatment with compounds **3b** and SC-560. In particular, COX-1 inhibition by compound **3b** reduced cell death induced by LPS almost to basal levels (Figure 5).

### 2.5. In Silico Prediction of the ADME Properties of Compounds 3

Compounds **3** were examined for their in silico ADME properties (Tables S1 and S2). In the initial stage, using SwissADME [53], we verified the absence of PAINs alerts from all compounds and their compliance with Lipinski’s rule. Moreover, compounds **3** were also examined with the aid of admetSAR [54], which predicted again a good drug-likeness in connection with their physicochemical properties, permeability through Caco2 cells, and hence a good capacity for intestinal absorption for all compounds, and a good blood-brain barrier penetration. All compounds were predicted not to interact with P-glycoprotein, which is a favorable feature in terms of drug distribution. On the other hand, all compounds were predicted to be potentially promiscuous inhibitors of CYP isoforms.

## 3. Materials and Methods

### 3.1. General Experimental Information

All reagents and solvents were of commercial quality and were used as received. Reactions were monitored by TLC analysis on Merck silica gel-G aluminum plates with fluorescent indicator. The starting anthranilic ester derivatives **1** were obtained using a literature protocol [30]. Melting points were measured in open capillary tubes and were uncorrected. A CEM Discover microwave synthesizer with a microwave power maximum level of 300 W and microwave frequency of 2455 MHz was employed for the microwave-assisted reactions. The ^1^H-NMR, ^13^C-NMR, and CH-correlation spectra were recorded on a Bruker Avance 250 MHz or 500 MHz NMR instrument (Rivas-Vaciamadrid, Spain) maintained by the CAI de Resonancia Magnética Nuclear, Universidad Complutense, using CDCl_3_, d_6_-DMSO or CD_3_OD as solvents and residual non-deuterated solvents as internal standards. The Mestrenova 10.0 (Mestrelab, Santiago de Compostela, Spain) software package was used throughout for data processing; chemical shifts are given in parts per million (δ-scale), and coupling constants are given in Hertz. Combustion microanalyses were performed by the CAI de Microanálisis Elemental, Universidad Complutense, on a Leco 932 CHNS analyzer. IR spectra were recorded on a Perkin Elmer (Tres Cantos, Spain) Paragon 1000 FT-IR instrument using thin films placed on a KBr disk, which were obtained by evaporation of the organic solvent solution of the compounds.

### 3.2. General Procedure for the Synthesis of Compounds 2 by Saponification of Anthranilic Esters 1

To a solution of the suitable anthranilate, ester **1** (1.2 mmol) was added a solution of aqueous sodium hydroxide 5 M (1 mL) in ethanol (10 mL). The reaction mixture was refluxed for 24 h, cooled, and diluted with H_2_O (10 mL). The solution was slowly neutralized, in an ice bath, with 0.5 M hydrochloric acid, until the appearance of a white precipitate, which was filtered and crystalized in ethanol to give compounds **2a**–**2g**.

*6-Amino-2,4-diphenylbenzoic acid* (**2a**). Prepared from ethyl 6-amino-2,4-diphenylbenzoate **1a** (305 mg, 1.2 mmol). Yield: 161 mg (58%), as a pale brown solid. Mp: 162–163 °C. Elemental analysis (%) calcd for C_19_H_15_NO_2_ (M = 289.33): C, 78.87; H, 5.23; N, 4.84; found: C, 78.83; H, 5.27; N, 4.82. IR ν_max_ (film): 3481, 3376, 3024, 1666, 1596, 1560 cm^−1^. ^1^H NMR (250 MHz, MeOD) δ 7.59 (d, *J* = 6.9 Hz, 2H), 7.46–7.23 (m, 10H), 7.03 (d, *J* = 1.7 Hz, 1H), 6.80 (d, *J* = 1.7 Hz, 1H). ^13^C NMR (63 MHz, MeOD) δ 173.1, 149.9, 145.3, 144.9, 144.3, 141.7, 129.8, 129.3, 128.9, 128.8, 128.0, 127.9, 119.4, 116.1, 114.7.

*6-Amino-2-phenyl-4-(4-tolyl)benzoic acid* (**2b**). Prepared from ethyl 6-amino-2-phenyl-4-(4-tolyl) benzoate **1b** (305 mg, 1.2 mmol). Yield: 218 mg (75%), as an off-white solid. Mp: 164–165 °C. Elemental analysis (%) calcd for C_20_H_17_NO_2_ (M = 303.35): C, 79.19; H, 5.65; N, 4.62; found: C, 79.23; H, 5.62; N, 4.58. IR ν_max_ (film): 3485, 3377, 3022, 2857, 1684, 1603 cm^−1^. ^1^H NMR (250 MHz, DMSO) δ 7.54 (d, *J* = 8.1 Hz, 2H), 7.44–7.32 (m, 5H), 7.27 (d, *J* = 8.4 Hz, 3H), 6.88 (d, *J* = 1.4 Hz, 1H), 2.33 (s, 3H). ^13^C NMR (63 MHz, DMSO) δ 169.6, 145.6, 143.2, 142.3, 142.2, 137.6, 136.5, 129.6, 128.1, 128.1, 127.0, 126.6, 118.7, 115.8, 114.3, 20.8.

*6-Amino-2-phenyl-4-(4-chlorophenyl)benzoic acid* (**2c**). Prepared from ethyl 6-amino-2-phenyl-4-(4-chlorophenyl) benzoate **1c** (338 mg, 1.2 mmol). Yield: 268 mg (69%), as a pale brown solid. Mp: 229–230 °C. Elemental analysis (%) calcd for C_19_H_14_ClNO_2_ (M = 323.77): C, 70.48; H, 4.36; N, 4.33; found: C, 70.53; H, 4.32; N, 4.29. IR ν_max_ (film): 3444, 3400, 2923, 2851, 1692 cm^−1^. ^1^H NMR (250 MHz, DMSO) δ 7.74–7.64 (m, 1H), 7.53 (d, *J* = 8.6 Hz, 1H), 7.45–7.32 (m, 3H), 7.29 (s, 1H), 6.96 (d, *J* = 1.3 Hz, 1H). ^13^C NMR (63 MHz, DMSO) δ 169.4, 150.9, 144.6, 143.2, 141.8, 141.1, 138.1, 133.0, 129.0, 128.6, 128.2, 128.1, 127.2, 119.3, 114.9.

*6-Amino-2,4-di-(4-chlorophenyl)benzoic acid* (**2d**). Prepared from ethyl 6-amino-2,4-di-(4-chlorophenyl) benzoate **1d** (371 mg, 1.2 mmol). Yield: 215 mg (50%), as a pale brown solid. Mp: 178–179 °C. Elemental analysis (%) calcd for C_19_H_13_Cl_2_NO_2_ (M = 358.22): C, 63.71; H, 3.66; N, 3.91; found: C, 63.68; H, 3.72; N, 3.86. IR ν_max_ (film): 3496, 3371, 1657, 1605, 1581, 1491 cm^−1^. ^1^H NMR (250 MHz, MeOD) δ 7.53 (d, *J* = 8.3 Hz, 2H), 7.42–7.33 (m, 3H), 7.33–7.23 (m, 4H), 6.99 (s, 1H), 6.70 (s, 1H). ^13^C NMR (63 MHz, MeOD) δ 172.1, 150.7, 144.7, 143.9, 143.1, 140.0, 135.0, 133.9, 130.8, 129.9, 129.5, 129.0, 118.8, 114.9, 114.5.

*6-Amino-2-phenyl-4-(4-methoxyphenyl)benzoic acid* (**2e**). Prepared from ethyl 6-amino-2-(4-methoxyphenyl)-2-phenylbenzoate **1e** (334 mg, 1.2 mmol). Yield: 238 mg (62%), as a pale-yellow solid. Mp: 195–196 °C. Elemental analysis (%) calcd for C_20_H_17_NO_3_ (M = 319.35): C, 75.22; H, 5.37; N, 4.39; found: C, 75.18; H, 5.35; N, 4.42. IR ν_max_ (film): 3484, 3372, 1647, 1605, 1581 cm^−1^. ^1^H NMR (250 MHz, DMSO) δ 7.58 (d, *J* = 8.7 Hz, 2H), 7.40–7.26 (m, 5H), 7.05–6.96 (m, 3H), 6.68 (d, *J* = 1.4 Hz, 1H), 3.79 (s, 3H). ^13^C NMR (63 MHz, DMSO) δ 170.2, 159.3, 149.0, 143.3, 142.8, 142.1, 132.0, 128.0, 128.0, 127.8, 126.8, 116.4, 114.4, 112.9, 112.3, 55.2.

*6-Amino-2,4-di-(4-bromophenyl)benzoic acid* (**2f**). Prepared from ethyl 6-amino-2,4-di-(4-bromophenyl) benzoate **1f** (456 mg, 1.2 mmol). Yield: 397 mg (74%), as an off-white solid. Mp: 239–240 °C. Elemental analysis (%) calcd for C_19_H_13_Br_2_NO_2_ (M = 447.12): C, 51.04; H, 2.93; N, 3.13; found: C, 51.07; H, 2.89; N, 3.18. IR ν_max_ (film): 2922, 2851, 1674, 1590, 1492 cm^−1^. ^1^H NMR (250 MHz, DMSO) δ 7.75–7.49 (m, 7H), 7.31 (d, *J* = 8.5 Hz, 3H), 6.93 (d, *J* = 1.2 Hz, 1H). ^13^C NMR (63 MHz, DMSO) δ 169.1, 150.9, 142.2, 141.3, 141.3, 138.3, 132.0, 131.0, 130.3, 129.4, 128.9, 121.8, 120.6, 113.8, 101.9.

*6-Phenylamino-2,4-diphenylbenzoic acid* (**2g**). Prepared from ethyl 6-phenylamino-2,4-diphenyl-2,3-dihydro benzoate **1g** (378 mg, 1.2 mmol). Yield: 333 mg (76%), as a pale-yellow solid. Mp: 182–183 °C. Elemental analysis (%) calcd for C_25_H_19_NO_2_ (M = 365.42): C, 82.17; H, 5.24; N, 3.83; found: C, 82.13; H, 5.29; N, 3.85. IR ν_max_ (film): 3374, 3055, 3029, 2924, 1656, 1589, 1557 cm^−1^. ^1^H NMR (250 MHz, CDCl_3_) δ 7.45 (d, *J* = 1.7 Hz, 1H), 7.41 (d, *J* = 1.4 Hz, 1H), 7.40 (d, *J* = 1.6 Hz, 1H), 7.33–7.23 (m, 7H), 7.23–7.16 (m, 4H), 7.12 (d, *J* = 7.9 Hz, 2H), 6.95 (t, *J* = 7.3 Hz, 1H), 6.90 (d, *J* = 1.5 Hz, 1H). ^13^C NMR (63 MHz, CDCl_3_) δ 174.56, 146.33, 145.39, 144.90, 142.57, 141.37, 140.10, 129.59, 128.90, 128.30, 128.16, 127.38, 127.30, 123.20, 121.48, 120.97, 113.01.

### 3.3. General Procedure for the Synthesis of m-Terphenylanilines 3 by Decarboxylation of Compounds 2

A tube containing a mixture of the appropriate anthranilic acid derivative (1.0 eq) and sodium hydroxide (1 eq) in DMF (2 mL) was sealed and placed in the cavity of a CEM Discover microwave oven. The tube was subjected to microwave irradiation, programmed at 180 °C and 200 W. After a period of 3–4 min, the temperature remained constant at 180 °C. After completion of the reaction (1.5 h), the tube was cooled to room temperature, and compounds **3** were precipitated by the addition of hexane in an ice bath and purified by recrystallization from ethanol.

*5′-Amino-m-terphenyl* (**3a**). Prepared from 6-amino-2,4-diphenylbenzoic acid **2a** (289 mg, 1.0 mmol). Yield: 243 mg (99%), as a white solid. Mp: 108–109 °C; lit [55], 107–109 °C. Elemental analysis (%) calcd for C_18_H_15_N (M = 245.32): C, 88.13; H, 6.16; N, 5.71; found: C, 88.16; H, 6.14; N, 5.65. IR ν_max_ (film): 3454, 3370, 3055, 1595. ^1^H NMR (250 MHz, DMSO) δ 7.64 (d, *J* = 7.1 Hz, 4H), 7.45 (t, *J* = 7.3 Hz, 4H), 7.35 (t, *J* = 7.2 Hz, 2H), 7.02 (s, 1H), 6.84 (d, *J* = 1.5 Hz, 2H), 5.32 (s, 2H). ^13^C NMR (63 MHz, DMSO) δ 149.6, 141.6, 141.1, 128.8, 127.3, 126.7, 113.3, 111.5. These characterization data were coincident with those found in the literature [28,56].

*4-Methyl-5′-amino-m-terphenyl* (**3b**). Prepared from 6-amino-2-phenyl-4-(4-tolyl) benzoic acid (303 mg, 1.0 mmol) **2b**. Yield: 257 mg (99%), as a white solid. Mp: 98–99 °C. Elemental analysis (%) calcd for C_19_H_17_N (M = 259.34): C, 87.99; H, 6.61; N, 5.40; found: C, 88.02; H, 6.63; N, 5.38. IR ν_max_ (film): 3418, 3294, 3025, 1594 cm^−1^. ^1^H NMR (250 MHz, CDCl_3_) δ 7.64 (d, *J* = 6.9 Hz, 2H), 7.55 (d, *J* = 8.1 Hz, 2H), 7.51–7.42 (m, 2H), 7.41–7.34 (m, 1H), 7.26 (s, 1H), 7.23 (t, *J* = 1.5 Hz, 1H), 6.93–6.89 (m, 2H), 2.43 (s, 3H). ^13^C NMR (63 MHz, DMSO) δ 149.6, 141.6, 141.5, 141.2, 138.2, 136.5, 129.4, 128.8, 127.3, 126.7, 126.5, 113.1, 111.3, 20.7. These characterization data were coincident with those found in the literature [28,55].

*4-Chloro-5′-amino-m-terphenyl* (**3c**). Prepared from 6-amino-2-phenyl-4-(4-chlorophenyl)benzoic acid **2c** (324 mg, 1.0 mmol). Yield: 201 mg, (72%), as a white solid. Mp: 96–97 °C. Elemental analysis (%) calcd for C_18_H_14_ClN (M = 279.76): C, 77.28; H, 5.04; N, 5.01; found: C, 77.25; H, 5.07; N, 5.04. IR ν_max_ (film): 3460, 3374, 3057, 2923, 1597, 1495 cm^−1^. ^1^H NMR (250 MHz, MeOD) δ 7.62 (d, *J* = 8.4 Hz, 4H), 7.45–7.38 (m, 4H), 7.36–7.30 (m, 1H), 7.12 (t, *J* = 1.6 Hz, 1H), 6.97 (t, *J* = 1.6 Hz, 1H), 6.95 (t, *J* = 1.6 Hz, 1H). ^13^C NMR (63 MHz, MeOD) δ 149.9, 144.1, 142.8, 142.6, 141.7, 134.2, 129.8, 129.7, 129.5, 128.3, 128.0, 116.7, 114.5, 113.9. These characterization data were coincident with those found in the literature [28,55].

*4,4″-Dichloro-5′-amino-m-terphenyl* (**3d**). Prepared from 6-amino-2,4-di-(4-chlorophenyl)benzoic acid (358 mg, 1.0 mmol) **2d**. Yield: 212 mg (99%), as an off-white solid. Mp: 253–254 °C. Elemental analysis (%) calcd for C_18_H_13_Cl_2_N (M = 314.21): C, 68.81; H, 4.17; N, 4.46; found: C, 68.86; H, 4.14; N, 4.42. IR ν_max_ (film): 3327, 3012, 2957, 1626, 1544, 1512 cm^−1^. ^1^H NMR (250 MHz, CDCl_3_) δ 7.60–7.51 (m, 4H), 7.43 (d, *J* = 8.7 Hz, 4H), 7.13 (t, *J* = 1.6 Hz, 1H), 6.88 (d, *J* = 1.6 Hz, 2H), 3.89 (bs, 2H). ^13^C NMR (63 MHz, CDCl_3_) δ 147.4, 142.0, 139.8, 133.6, 129.0, 128.5, 116.6, 113.0. These characterization data were coincident with those found in the literature [28].

*4-Methoxy-5′-amino-m-terphenyl* (**3e**). Prepared from 6-amino-2-(4-methoxyphenyl)-2-phenylbenzoic acid **2e** (319 mg, 1.0 mmol). Yield: 215 mg (78%), as a white solid. Mp: 118–119 °C. Elemental analysis (%) calcd for C_19_H_17_NO (M = 275.34): C, 82.88; H, 6.22; N, 5.09; found: C, 82.83; H, 6.26; N, 5.04. IR ν_max_ (film): 3447, 3369, 3030, 1596, 1514 cm^−1^. ^1^H NMR (250 MHz, DMSO) δ 7.63 (d, *J* = 7.2 Hz, 2H), 7.58 (d, *J* = 8.7 Hz, 2H), 7.44 (t, *J* = 7.3 Hz, 2H), 7.34 (t, *J* = 7.2 Hz, 1H), 7.06–6.95 (m, 3H), 6.80 (s, 2H), 5.28 (br s, 2H), 3.79 (s, 3H). ^13^C NMR (63 MHz, DMSO) δ 158.8, 149.6, 141.6, 141.3, 141.2, 133.4, 128.8, 127.7, 127.2, 126.7, 114.2, 112.9, 111.1, 110.9, 55.2. These characterization data were coincident with those found in the literature [28,55].

*4,4″-Dibromo-5′-amino-m-terphenyl* (**3f**). Prepared from 6-amino- 2,4-di-(4-bromophenyl)benzoic acid **2f** (447 mg, 1.0 mmol). Yield: 294 mg (73%), as an off-white solid. Mp: 163–164 °C. Elemental analysis (%) calcd for C_18_H_13_Br_2_N (M = 403.11): C, 53.63; H, 3.25; N, 3.47; found: C, 53.61; H, 3.26; N, 3.43. IR ν_max_ (film): 3393, 3308, 1598, 1491 cm^−1^. ^1^H NMR (250 MHz, DMSO) δ 7.69–7.54 (m, 8H), 7.01 (s, 1H), 6.84 (d, *J* = 1.4 Hz, 2H), 5.38 (br s, 2H). ^13^C NMR (63 MHz, DMSO) δ 149.9, 140.4, 140.1, 131.7, 128.8, 120.7, 112.9, 111.5.

*5′-Phenylamino-m-terphenyl* (**3g**). Prepared from 6-phenylamino- 2,4-diphenylbenzoic acid **2g** (365 mg, 1.0 mmol). Yield: 244 mg, (76%), as an off-white solid. Mp: 128–129 °C. Elemental analysis (%) calcd for C_24_H_19_N (M = 321.41): C, 89.68; H, 5.96; N, 4.36; found: C, 89.62; H, 5.93; N, 4.37. IR ν_max_ (film): 3401, 3050, 3026, 1594, 1533 cm^−1^. ^1^H NMR (250 MHz, CDCl_3_) δ 7.55 (d, *J* = 1.6 Hz, 1H), 7.51 (d, *J* = 6.9 Hz, 2H), 7.44–7.29 (m, 7H), 7.29–7.20 (m, 3H), 7.19–7.11 (m, 3H), 7.01 (d, *J* = 7.5 Hz, 2H), 6.85 (t, *J* = 7.3 Hz, 1H), 5.61 (br s, 1H). ^13^C NMR (63 MHz, CDCl_3_) δ 143.4, 141.5, 141.0, 140.6, 138.8, 131.5, 130.6, 129.6, 129.5, 129.1, 128.9, 127.7, 127.5, 127.2, 121.4, 120.0, 118.4, 116.1.

General procedure for the synthesis of urea derivatives **4** and **5**. To a stirred solution of the suitable *m*-terphenylamine **4** (0.7 equiv) or compound **1e** in anhydrous dichloromethane (5 mL), under an argon atmosphere, was added the suitable isocyanate (1.0 equiv) and the reaction mixture was stirred for 12 to 18 h at room temperature. Upon completion (TLC), the reaction mixture was diluted with dichloromethane (100 mL), which was washed with H_2_O (20 mL) and a saturated aqueous solution of NaCl (20 mL), dried over anhydrous Na_2_SO_4_, filtered, and concentrated in vacuo to give a residue that was purified by flash chromatography on silica gel eluting with DCM/petroleum ether (4:1), to give the expected compounds.

*1-([m-Terphenyl]-5′-yl)-3-cyclopentyl urea* (**4a**). Prepared from 5′-amino-*m*-terphenyl **3a** (172 mg, 0.7 mmol) and cyclopentyl isocyanate (0.12 mL, 1.05 mmol). Yield: 217 mg (87%), as a white solid. Mp: 224–225 °C. Elemental analysis (%) calcd for C_24_H_24_N_2_O (M = 356.46): C, 80.87; H, 6.79; N, 7.86; found: C, 80.92; H, 6.83; N, 7.81. IR ν_max_ (film): 3337, 2919, 2851, 1643, 1559 cm^−1^. ^1^H NMR (250 MHz, DMSO) δ 8.50 (s, 1H), 7.69 (d, *J* = 7.7 Hz, 6H), 7.48 (t, *J* = 7.3 Hz, 4H), 7.42–7.34 (m, 3H), 6.28 (d, *J* = 7.1 Hz, 1H), 4.05–3.87 (m, 1H), 1.93–1.77 (m, 2H), 1.73–1.49 (m, 4H), 1.47–1.32 (m, 2H). ^13^C NMR (63 MHz, DMSO) δ 154.9, 141.7, 141.4, 140.5, 129.0, 127.6, 126.9, 118.2, 115.1, 51.0, 32.8, 23.2. These characterization data were coincident with those found in the literature [27].

*1-([4-Methyl-m-terphenyl]-5′-yl)-3-cyclopentyl urea* (**4b**). Prepared from 4-methyl-5′-amino-*m*-terphenyl **3b** (182 mg, 0.7 mmol) and cyclopentyl isocyanate (0.12 mL, 1.05 mmol). Yield: 254 mg (98%), as an off-white solid. Mp: 217–218 °C. Elemental analysi**s** (%) calcd for C_25_H_26_N_2_O (M = 370.49): C, 81.05; H, 7.07; N, 7.56; found: C, 81.02; H, 7.11; N, 7.53. IR ν_max_ (film): 3314, 2955, 2857, 1640, 1557 cm^−1^. ^1^H NMR (250 MHz, DMSO) δ 8.47 (s, 1H), 7.68 (d, *J* = 7.1 Hz, 2H), 7.64 (d, *J* = 1.5 Hz, 2H), 7.58 (d, *J* = 8.1 Hz, 2H), 7.48 (t, *J* = 7.3 Hz, 2H), 7.42–7.34 (m, 2H), 7.28 (d, *J* = 8.0 Hz, 2H), 6.26 (d, *J* = 7.1 Hz, 1H), 4.06–3.87 (m, 1H), 1.93–1.77 (m, 2H), 1.72–1.49 (m, 4H), 1.39 (dd, *J* = 11.5, 6.0 Hz, 2H). ^13^C NMR (63 MHz, DMSO) δ 154.9, 141.6, 141.4, 141.3, 140.6, 137.6, 136.9, 129.5, 128.9, 127.6, 126.9, 126.7, 117.9, 114.8, 51.0, 32.9, 23.2, 20.7.

*1-([4-Chloro-m-terphenyl]-5′-yl)-3-cyclopentylurea* (**4c**). Prepared from 4-chloro-5′-amino-*m*-terphenyl (196 mg, 0.7 mmol) and cyclopentyl isocyanate (0.12 mL, 1.05 mmol). Yield: 178 mg (65%), as a white solid. Mp: 203–204 °C. Elemental analysis (%) calcd for C_24_H_23_ClN_2_O (M = 390.91): C, 73.74; H, 5.93; N, 7.17; found: C, 73.71; H, 6.02; N, 7.15. IR ν_max_ (film): 3323, 2919, 2851, 1639, 1556 cm^−1^. ^1^H NMR (250 MHz, DMSO) δ 8.34 (s, 1H), 7.55 (dd, *J* = 14.6, 5.9 Hz, 6H), 7.35 (dd, *J* = 16.4, 8.1 Hz, 4H), 7.29–7.18 (m, 2H), 6.13 (d, *J* = 7.1 Hz, 1H), 3.89–3.72 (m, 1H), 1.78–1.62 (m, 2H), 1.56–1.33 (m, 4H), 1.32–1.14 (m, 2H). ^13^C NMR (63 MHz, DMSO) δ 154.9, 141.7, 141.5, 140.3, 140.0, 139.3, 132.4, 129.0, 128.9, 128.7, 127.7, 126.9, 118.1, 115.4, 114.9, 51.0, 32.8, 23.2.

*1-([4,4″-Dichloro-m-terphenyl]-5′-yl)-3-cyclopentylurea* (**4d**). Prepared from 4,4**″**-dichloro-5′-amino-*m*-terphenyl (220 mg, 0.7 mmol) and cyclopentyl isocyanate (0.12 mL, 1.05 mmol). Yield: 179 mg (60%), as a pale-yellow solid. Mp: 239–240 °C. Elemental analysis (%) calcd for C_24_H_22_Cl_2_N_2_O (M = 425.35): C, 67.77; H, 5.21; N, 6.59; found: C, 67.82; H, 5.19; N, 6.63. IR ν_max_ (film): 3258, 3092, 2955, 1728, 1633 cm^−1^. ^1^H NMR (250 MHz, DMSO) δ 8.42 (s, 1H), 7.63 (d, *J* = 8.6 Hz, 4H), 7.59 (d, *J* = 1.5 Hz, 2H), 7.43 (d, *J* = 8.5 Hz, 4H), 7.33 (d, *J* = 2.0 Hz, 2H), 6.21 (d, *J* = 7.1 Hz, 1H), 3.94–3.78 (m, 1H), 1.86–1.67 (m, 2H), 1.62–1.39 (m, 4H), 1.37–1.20 (m, 2H). ^13^C NMR (63 MHz, DMSO) δ 155.2, 142.2, 140.5, 139.5, 132.8, 129.2, 129.0, 118.3, 115.6, 51.3, 33.2, 23.6.

*1-([4-Methoxy-m-terphenyl]-5′-yl)-3-cyclopentylurea* (**4e**). Prepared from 4-methoxy-5′-amino-*m*-terphenyl (193 mg, 0.7 mmol) and cyclopentyl isocyanate (0.12 mL, 1.05 mmol). Yield: 170 mg (63%), as a white solid. Mp: 208–209 °C. Elemental analysis (%) calcd for C_25_H_26_N_2_O_2_ (M = 386.49): C, 77.69; H, 6.78; N, 7.25; found: C, 77.73; H, 6.82; N, 7.21. IR ν_max_ (film): 3312, 2954, 1640, 1605, 1556 cm^−1^. ^1^H NMR (250 MHz, MeOD) δ 7.66 (t, *J* = 1.8 Hz, 1H), 7.63 (d, *J* = 1.1 Hz, 1H), 7.61 (d, *J* = 2.1 Hz, 1H), 7.60–7.54 (m, 3H), 7.48–7.42 (m, 1H), 7.40 (dd, *J* = 3.4, 1.8 Hz, 2H), 7.38–7.30 (m, 1H), 4.16–4.01 (m, 1H), 3.83 (s, 3H), 2.08–1.90 (m, 2H), 1.82–1.58 (m, 4H), 1.58–1.42 (m, 2H). ^13^C NMR (63 MHz, MeOD) δ 160.9, 158.0, 143.7, 143.4, 142.6, 141.9, 134.8, 129.8, 129.1, 128.5, 128.1, 120.4, 117.0, 116.9, 115.2, 55.8, 52.9, 34.2, 24.6.

*1-([4,4″-Dibromo-m-terphenyl]-5′-yl)-3-cyclopentylurea* (**4f**). Prepared from 4,4**″**-dibromo-5′-amino-*m*-terphenyl (282 mg, 0.7 mmol) and cyclopentyl isocyanate (0.12 mL, 1.05 mmol). Yield: 259 mg (72%), as an off-white solid. Mp: 222–223 °C. Elemental analysis (%) calcd for C_24_H_22_Br_2_N_2_O (M = 514.25): C, 56.05; H, 4.31; N, 5.45; found: C, 56.07; H, 4.35; N, 5.42. IR ν_max_ (film): 3294, 2955, 2860, 1631, 1557 cm^−1^. ^1^H NMR (250 MHz, DMSO) δ 8.51 (s, 1H), 7.75–7.59 (m, 10H), 7.42 (s, 1H), 6.30 (d, *J* = 7.1 Hz, 1H), 4.03–3.88 (m, 1H), 1.93. ^13^C NMR (63 MHz, DMSO) δ 154.9, 141.9, 140.2, 139.5, 131.8, 129.0, 121.1, 117.9, 115.2, 51.0, 32.8, 23.2.

*Ethyl 6-(3′-cyclohexylureido)-2-(4-methoxyphenyl)-4-phenylbenzoate* (**5**). Prepared from ethyl-6-amino-2-(4-methoxyphenyl)-2-phenylbenzoate (243 mg, 0.7 mmol) and cyclohexyl isocyanate (0.13 mL, 1.05 mmol). Yield: 208 mg (63%), as a white solid. Mp: 234–235 °C. Elemental analysis (%) calcd for C_29_H_32_N_2_O_4_ (M = 472.58): C, 73.70; H, 6.83; N, 5.93; found: C, 73.67; H, 6.84; N, 5.90. IR ν_max_ (film): 3302, 2926, 2850, 1691, 1644, 1542 cm^−1^. ^1^H NMR (250 MHz, CDCl_3_) δ 8.95 (bs, 1H), 8.60 (d, *J* = 1.8 Hz, 1H), 7.66 (d, *J* = 8.8 Hz, 2H), 7.46–7.30 (m, 5H), 7.23 (d, *J* = 1.8 Hz, 1H), 6.97 (d, *J* = 8.8 Hz, 2H), 3.93 (q, *J* = 7.2 Hz, 2H), 3.87 (s, 3H), 3.68 (t, *J* = 10.5 Hz, 1H), 2.12–1.99 (m, 2H), 1.85–1.60 (m, 3H), 1.53–1.33 (m, 2H), 1.30–1.11 (m, 3H), 0.73 (t, *J* = 7.2 Hz, 3H). ^13^C NMR (63 MHz, CDCl_3_) δ 170.4, 160.2, 155.1, 154.7, 144.4, 144.2, 143.4, 134.6, 132.6, 129.0, 128.5, 128.5, 127.4, 122.8, 116.6, 114.6, 68.2, 61.5, 55.8, 34.1, 26.0, 25.4, 13.4.

### 3.4. Docking Studies

The structure of crystallized COX-1 was obtained from the RCSB Protein Data Bank (PDBid: 5WBE) [34]. Water molecules and cocrystallized mofezolac were removed, and AM1-BCC (AMBER ff14SB) charges [57] were added to the protein, which was further minimized using the Antechamber module of UCSF Chimera [58]. The resultant structure was minimized using the same program. Ligands were prepared using a similar protocol from the SMILES code. Docking calculations were performed twice. For obtaining the initial geometry, all ligands were docked using VINA [59] and a search area which included the binding area itself and surroundings (center: x = 36.787, y = 162.678, z = 28.600; dimensions: x = 30, y = 30, z = 30; grid unit = 1 Å). The best-ranked conformations were redocked using AutoDock [60] in order to obtain more accurate binding energy. There were used maps of the same search area to obtain final binding poses and their associated binding energies.

### 3.5. Cyclooxygenase Colorimetric Inhibitor Screening Assay

Newly synthesized compounds were evaluated for their ability to inhibit ovine COX-1 or human COX-2 enzyme by measuring the extent (%) of enzyme activity inhibition at different concentrations (0.001, 0.01, 0.1, 1, 10, 50 μM). The inhibition of the enzyme was evaluated by using a colorimetric COX inhibitor screening assay kit (Catalog No. 701050, Cayman Chemicals, Ann Arbor, MI, USA) following the manufacturer’s instructions. The assay kit measures the peroxidase component of COXs that is assayed colorimetrically by monitoring the appearance of oxidized N,N,N′,N′-tetramethyl-*p*-phenylenediamine (TMPD) a λ = 590 nm. COX is a bifunctional enzyme exhibiting both cyclooxygenase and peroxidase activities. The cyclooxygenase component catalyzes the conversion of arachidonic acid into the hydroperoxide PGG_2_, and then the peroxidase component catalyzes PGG_2_ reduction into the corresponding alcohol PGH_2_, the precursor of PGs, thromboxane, and prostacyclin. Stock solutions of test compounds were dissolved in DMSO.

### 3.6. Microglia BV2 Cell Line

BV-2 cells were maintained in Roswell Park Memorial Institute (RPMI) medium 1640 supplemented with 10% FBS and antibiotics (penicillin 100 U/mL, streptomycin 100 μg/mL). Cultures were seeded into flasks containing supplemented medium and kept at 37 °C in a humidified atmosphere of 5% CO_2_ and 95% air. For assays, BV-2 cells were subcultured in 48-well plates at a seeding density of 1 × 10^5^ cells per well. Cells were treated with LPS (Sigma-Aldrich, Madrid, Spain) for 24 or 48 h before confluence in RPMI with 1% FBS.

### 3.7. Griess Reaction

The NO assay was performed using the previously reported protocols. Briefly, 100 μL of culture supernatants were mixed with the same volume of Griess assay regent (1% sulfanilamide (Sigma Aldrich, Madrid, Spain), 0.1% naphthylethylenediamine dihydrochloride, and 2.5% phosphoric acid). Ten minutes later, the 560-nm absorbance was measured.

### 3.8. ROS Measurement

To measure cellular ROS, we used the molecular probe 2′,7′-dichlorodihydrofluorescein diacetate (H2DCFDA). Glial cells were loaded with 5 µM H2DCFDA as it was previously described [61] (Parada et al., 2015). Fluorescence was measured in a fluorescence microplate reader (FLUOstar Galaxy; BMG Labtech, Offenburg, Germany). Wavelengths of excitation and emission were 485 and 520 nm, respectively. The relative pixel intensity was measured in identical regions with (ImageJ 1.52a software, National Institutes of Health, Bethesda, MD, USA).

### 3.9. Preparation of Organotypic Hippocampal Cultures (OHCs)

OHCs were conducted on 8–10-day-old Sprague-Dawley rats supplied by the animal facilities of Universidad Autónoma de Madrid (Madrid, Spain). Cultures were prepared according to the methods described by Stoppini et al. [52] with some modifications. Briefly, 300-µm-thick hippocampal slices were prepared from rat pups using a McIlwain tissue chopper and separated in ice-cold Hank’s balanced salt solution (HBSS) composed of (mM): glucose 15, CaCl_2_ 1.3, KCl 5.36, NaCl 137.93, KH_2_PO_4_ 0.44, Na_2_HPO_4_ 0.34, MgCl_2_ 0.49, MgSO_4_ 0.44, NaHCO_3_ 4.1, and HEPES 25; 100 U/mL penicillin and 0.100 mg/mL gentamicin. Approximately 4–6 slices were placed on Millicell 0.4-µm culture insert (Millipore, Burlington, MA, USA) within each well of a six-well culture tray with the medium, where they remained for 7 days. The culture medium, which consisted of 50% minimal essential medium, 25% HBSS, and 25% heat-inactivated horse serum, was purchased from Life Technologies (Madrid, Spain). The medium was supplemented with 3.7 mg/mL d-glucose, 2 mmol/L l-glutamine, 2% of B-27 Supplement Minus antioxidants (Life Technologies, Madrid, Spain), and 100 U/mL penicillin. OHCs were cultivated in a humidified atmosphere at 37 °C and 5% CO_2_, and the medium was changed twice a week.

### 3.10. Quantification of Cell Death in OHCs: Propidium Iodide (PI) Uptake

Cell death was determined in the CA1 region by staining the OHCs with propidium iodide (PI). Thirty minutes before analyzing fluorescence, slices were incubated with PI (1 µg/mL). Fluorescence was measured in an epifluorescence Olympus BX50WI microscope. The wavelengths of excitation and emission for PI were 530 and 580, respectively. Images were taken at magnifications of 2×. Fluorescence was measured in each slice using ImageJ.

## 4. Conclusions

In conclusion, *m*-terphenylamine derivatives are selective COX-1 inhibitors and are able to block microglial inflammatory and oxidative response, reducing nitrite release and ROS overproduction. One of these compounds (**3b**) was further studied and exhibited an interesting neuroprotective profile in organotypic hippocampal cultures subjected to LPS. Moreover, our data support the participation of COX-1 in the proinflammatory cascade induced by LPS not only in preventing microglia activation but also in the subsequent neuronal cell death, and therefore its COX-1 inhibition is a promising approach to provide enhanced neuroprotection against acute inflammatory processes.

## Figures and Tables

**Table 1:** Cyclooxygenase inhibition data.

Entry	Compound	*o*COX-1; IC_50_ (µM) (% Inhibition at 50 µM) ^a^	*h*COX-2; IC_50_ (µM) (% Inhibition at 50 µM)
1	Mofezolac	0.023 ± 0.0013 (100)	>50 (42)
2	Celecoxib	>50 (40)	0.24 (95)
3	**1a**	>50	>50
4	**1b**	>50	NM (6)
5	**1c**	NM (11)	>50
6	**1d**	NM (11)	NM (5)
7	**1e**	NM (9)	>50
8	**1f**	>50	>50
9	**1g**	>50	>50
10	**2a**	NM (32)	NM (3)
11	**2b**	NM (46)	NM (2)
12	**2c**	>50	NM (4)
13	**2d**	>50	NM (13)
14	**2e**	>50	NM (20)
15	**2f**	NM (8)	NM (12)
16	**2g**	NM (7)	NM (13)
17	**3a**	0.80 ± 0.39 (86)	5.4 (70)
18	**3b**	1.10 ± 0.18 (83)	>50
19	**3c**	4.70 ± 3.04 (100)	>50
20	**3d**	NM (36]	>50
21	**3e**	0.90 ± 0.76 (73)	NM (8)
22	**3f**	>50	>50
23	**3g**	>50	>50
24	**4a**	>50	>50
25	**4b**	>50	>50
26	**4c**	>50	>50
27	**4d**	>50	>50
28	**4e**	>50	>50
29	**4f**	NM (10)	NM (30)
30	**5**	>50	>50

^a^ NM: not measured.

**Table 2:** Binding energies of binding modes in Figure 2 below for the reference compounds mofezolac and celecoxib and the four active compounds **3**.

Compound	Binding Energy (kcal/mol)
Mofezolac	7.97
Celecoxib	5.06
**3a**	6.68
**3b**	6.68
**3c**	6.54
**3e**	5.54

**Table 3:** Effect of COX-1 inhibitors **3** on nitrite release, measured as % of NO release, in BV2 microglia cells subjected to LPS for 24 and 48 h. Data are expressed as means ± SEM of at least four different cell batches in triplicate. ^###^ *p* < 0.001 in comparison with non-treated cells, * *p* < 0.05, ** *p* < 0.01, *** *p* < 0.001 with respect to LPS-treated cells.

Treatment	Concentration	Nitrite Release after 24 h	Nitrite Release after 48 h
Basal	0	100	100
LPS	1 μg/mL	150 ± 8 ^###^	188 ± 15 ^###^
SC-560	100 nM	114 ± 9 *	135 ± 8 *
**3a**	1 μM	130 ± 14	145 ± 17
**3b**	1 μM	123 ± 8	140 ± 14
**3c**	1 μM	135 ± 6	181 ± 9
**3e**	1 μM	124 ± 9	144 ± 15
**3a**	3 μM	110 ± 7 *	119 ± 10 **
**3b**	3 μM	100 ± 4 ***	123 ± 10 **
**3c**	3 μM	117 ± 8	163 ± 5
**3e**	3 μM	110 ± 6 **	133 ± 13 *
**3a**	10 μM	122 ± 10	120 ± 15 **
**3b**	10 μM	105 ± 6 **	128 ± 10 *
**3c**	10 μM	110 ± 6 *	138 ± 7
**3e**	10 μM	103 ± 9 **	131 ± 13 *

## Data Availability

Data are contained within the article or Supplementary Material.

## Supplementary Materials

The following are available online at https://www.mdpi.com/article/10.3390/molecules28145374/s1, ADME prediction data and copies of spectra. References [62,63,64,65,66] are cited in the Supplementary Materials.

## Sample Availability

Samples of the compounds are available from the authors.
