# Supplementary material for: m-Terphenylamines, Acting as Selective COX-1 Inhibitors, Block Microglia Inflammatory Response and Exert Neuroprotective Activity
Source: Molecules. 2023 Jul 13;28(14):5374. doi: 10.3390/molecules28145374 (PMC10384011; doi:10.3390/molecules28145374)

Supporting Information

m-Terphenylamines, acting as selective COX-1 inhibitors, block microglia inflammatory response and exert neuroprotective activity

Damiano Rocchi [1], Juan F. González [1], Olmo Martín-Cámara [1], Maria Grazia Perrone [2], Morena Miciaccia [2],
Antonio Scilimati [2], Celine Decouty-Pérez [3], Esther Parada [3], Javier Egea [3,*] and J. Carlos Menéndez [1,*]

[1] Unidad de Química Orgánica y Farmacéutica, Departamento de Química en Ciencias Farmacéuticas,
Facultad de Farmacia, Universidad Complutense, 28040 Madrid, Spain; rocchid83@gmail.com (D.R.);
jfgonzal@ucm.es (J.F.G.); olmomart@ucm.es (O.M.-C.)

[2] Dipartimento di Farmacia—Scienze del Farmaco, Università degli Studi di Bari Aldo Moro, 70121 Bari, Italy; mariagrazia.perrone@uniba.it (M.G.P.); morena.miciaccia@uniba.it (M.M.); antonio.scilimati@uniba.it (A.S.)

[3] Molecular Neuroinflammation and Neuronal Plasticity Research Laboratory, Hospital Universitario Santa Cristina, Instituto de Investigación Sanitaria-Hospital Universitario de la Princesa, 28009 Madrid, Spain; celinedecouty96@gmail.com (C.D.-P.); esther.parada@educa.madrid.org (E.P.)

* Correspondence: javier.egea@inv.uam.es (J.E.); josecm@ucm.es (J.C.M.)

Content:

Prediction of physicochemical and ADMET properties of compounds **3** S2

Copies of NMR spectra S4

Prediction of ADME properties of compounds 3

Table S1. Swiss-ADME characterization of drug-like properties of compounds **3**

Smiles code	Cmpd Number	Rotable bonds	TPSA[1]	Log P o/w [2]	Log S[3]	Predicted GI Absorption[4]	Lipinski violations[5]	PAINS alerts[6]
NC1=CC(C2=CC=CC=C2)=CC(C3=CC=CC=C3)=C1	**3a**	2	26.02	4.08	-4.76 (M)	High	1	0
NC1=CC(C2=CC=C(C)C=C2)=CC(C3=CC=CC=C3)=C1	**3b**	2	26.02	4.42	-5.04 (M)	High	1	0
NC1=CC(C2=CC=C(Cl)C=C2)=CC(C3=CC=CC=C3)=C1	**3c**	2	26.02	4.71	-5.62 (M)	High	1	0
NC1=CC(C2=CC=C(Cl)C=C2)=CC(C3=CC=C(Cl)C=C3)=C1	**3d**	2	26.02	5.15	-5.91 (P)	High	1	0
NC1=CC(C2=CC=C(OC)C=C2)=CC(C3=CC=CC=C3)=C1	**3e**	3	35.25	4.06	-4.79 (M)	High	0	0
NC1=CC(C2=CC=C(Br)C=C2)=CC(C3=CC=C(Br)C=C3)=C1	**3f**	2	26.02	5.32	-6.55 (P)	High	1	0
C1(C2=CC=CC=C2)=CC(C3=CC=CC=C3)=CC(NC4=CC=CC=C4)=C1	**3g**	4	12.03	5.73	-6.51 (P)	Low	1	0

1. Topological Polar Surface Area calculated from: Etrl, P.; Rohde, B.; Selzer, P. Fast calculation of molecular polar surface area as a sum of fragment-based contributions and its application to the prediction of drug transport properties. *J. Med. Chem*. **2000**, *43*, 3714–3717. 2.
2. Consensus Log P o/w average of 5 prediction methods.
3. ESOL topological method implemented from Delaney, J. S. Prediction of aqueous solubility and partition coefficient optimized by a genetic algorithm-based descriptor selection method. *J. Chem. Inf. Model*. **2004**, *44*, 1000-1005. M, moderately soluble. P, poorly soluble.
4. Saina, A.; Zoete, V. A BOILED-Egg to predict gastrointestinal absorption and brain penetration of small molecules. *Chem. Med. Chem*. **2016**, *11*, 1117-1121.
5. Lipinski, C. A.; Lombardo, F.; Dominy, B. W.; Feeney, P. J. Experimental and computational approaches to estimate solubility and permeability in drug discovery and development settings. *Adv. Drug Deliv. Rev*. **2001**, *46*, 3-26. Compounds with no or only one violation are considered to comply with Lipinski's rule.
6. Baell, J.B.; Holloway G. A. New substructure filters for removal of pan assay interference compounds (PAINS) from screening libraries and for their exclusion in bioassays. *J. Med. Chem*. **2010**, *53*, 2719-2740.

Table S2. Admet-SAR prediction of ADME properties of compounds 3

		**Absorption**		**Distribution**			**Metabolism**
	Physicochemical-drug-likeness[1]	Human intestinal absorption	Caco-2 Permeability	Blood-Brain Barrier (BBB) penetration	P-glycoprotein substrate	P-glycoprotein inhibition	CYP inhibitory promiscuity[2]
**3a**	+	+ (0.99)	+ (0.87)	+ (0.97)	- (0.97)	- (0.89)	+ (0.76)
**3b**	+	+ (0.99)	+ (0.88)	+ (0.97)	- (0.96)	- (0.73)	+ (0.70)
**3c**	+	+ (0.99)	+ (0.83)	+ (0.97)	- (0.98)	- (0.85)	+ (0.76)
**3d**	+	+ (0.99)	+ (0.82)	+ (0.97)	- (0.98)	- (0.83)	+ (0.76)
**3e**	+	+ (0.99)	+ (0.91)	+ (0.70)	- (0.94)	- (0.64)	+ (0.76)
**3f**	+	+ (0.99)	+ (0.82)	+ (0.97)	- (0.98)	- (0.81)	+ (0.79)
**3g**	+	+ (0.99)	+ (0.87)	+ (0.95)	- (0.98)	- (0.79)	+ (0.85)

1 Molecular weight, alogP, number of atoms, number of rings, H-bond acceptors, and H-bond donors was used to calculate this factor.

2 CYP inhibitory promiscuity refers to the capacity for compounds to bind to different CYP enzymes (substrate of CYP2C9, 2D6, 3A4 and inhibitor of CYP1A2, 2D6, 2C9, 2C19, 3A4).

Copies of NMR spectra

6-Amino-2,4-diphenylbenzoic acid (2a)

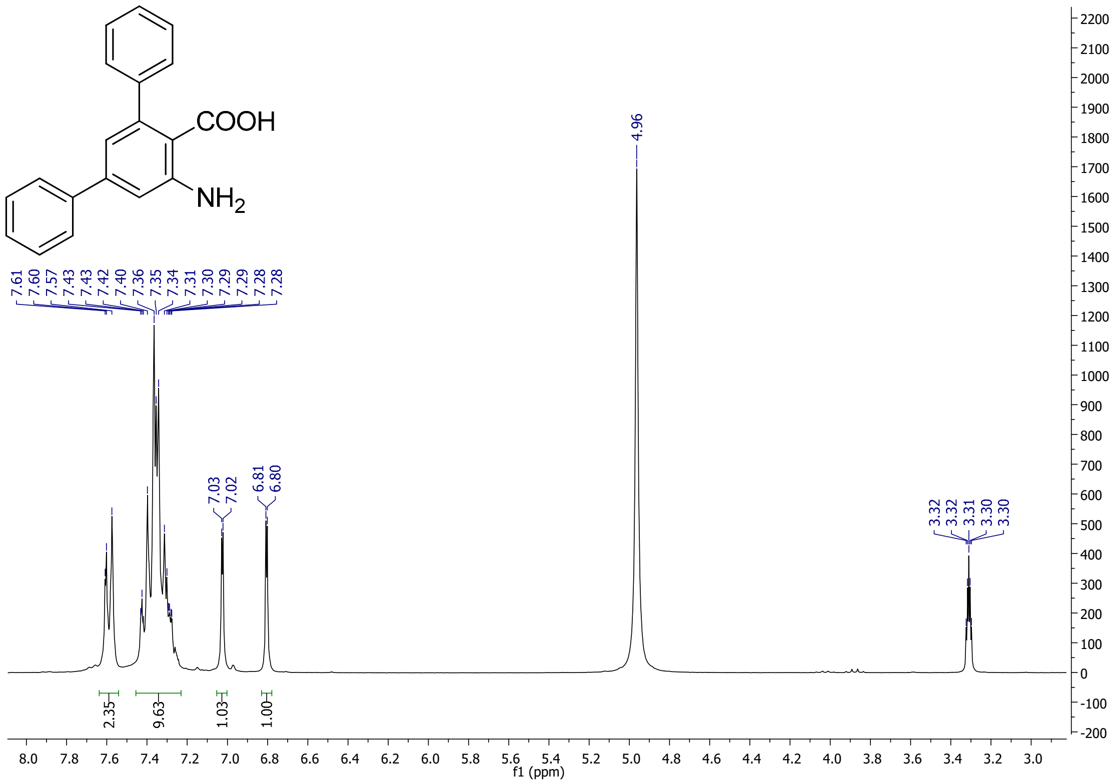

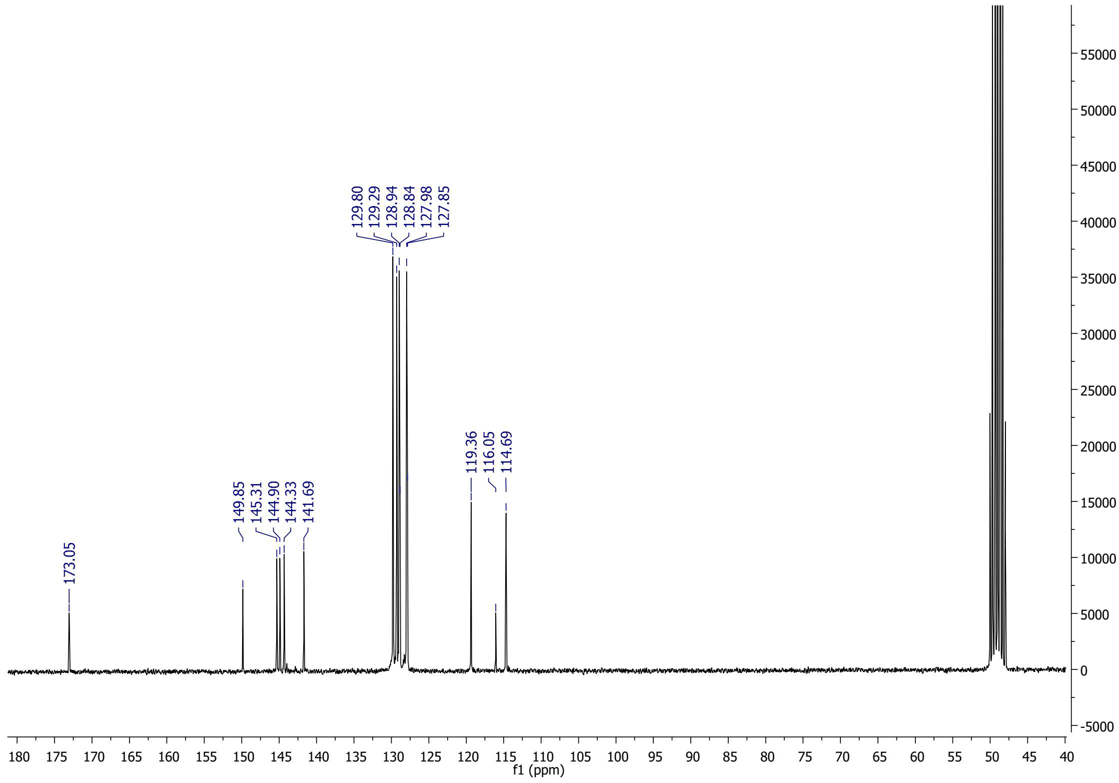

6-Amino-2-phenyl-4-(4-tolyl)benzoic acid (2b)

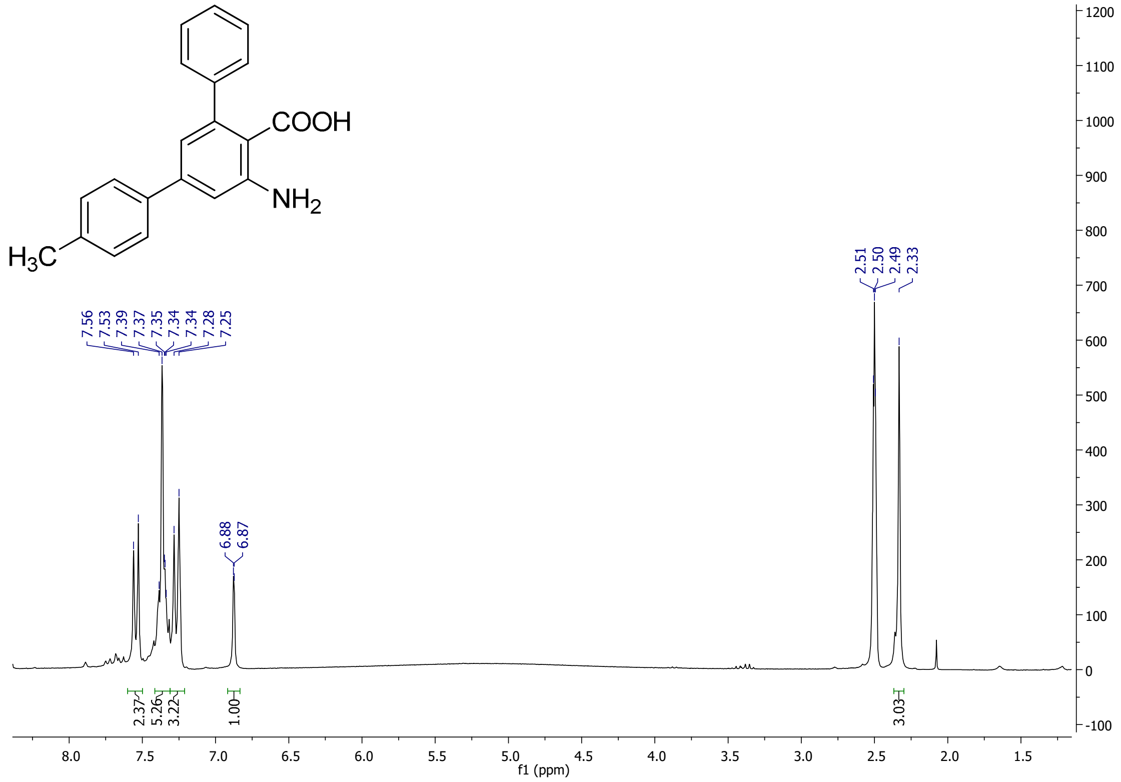

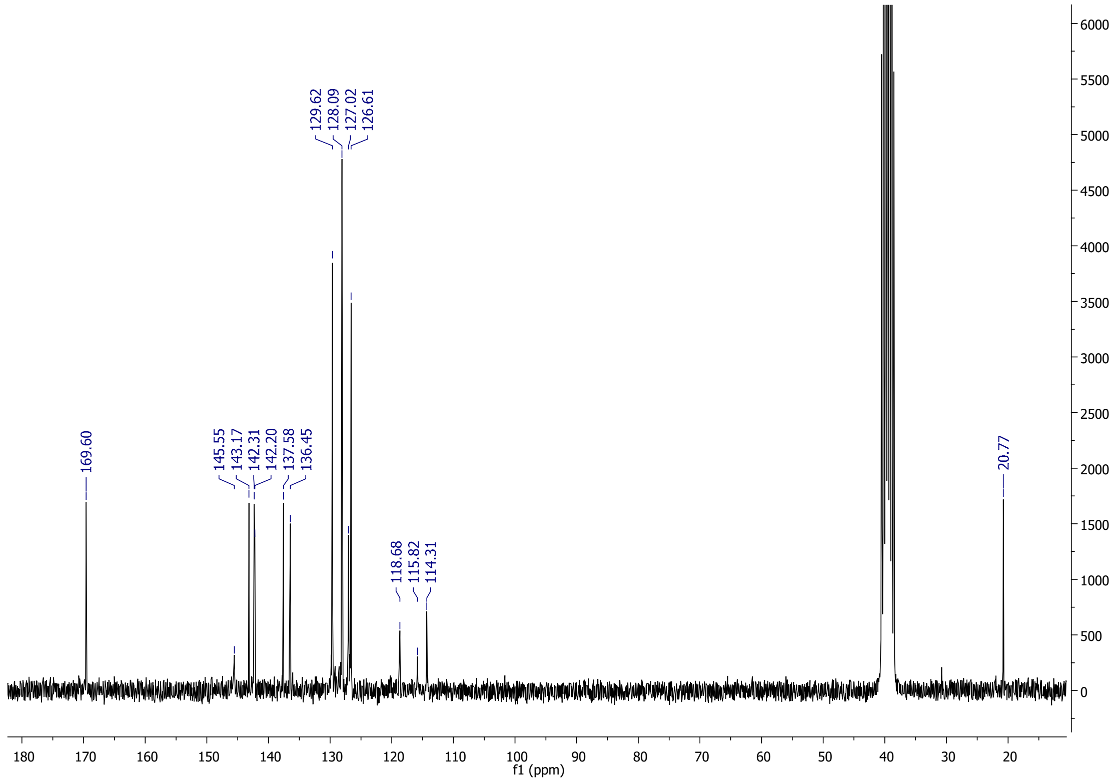

6-Amino-2-phenyl-4-(4-chlorophenyl)benzoic acid (2c)

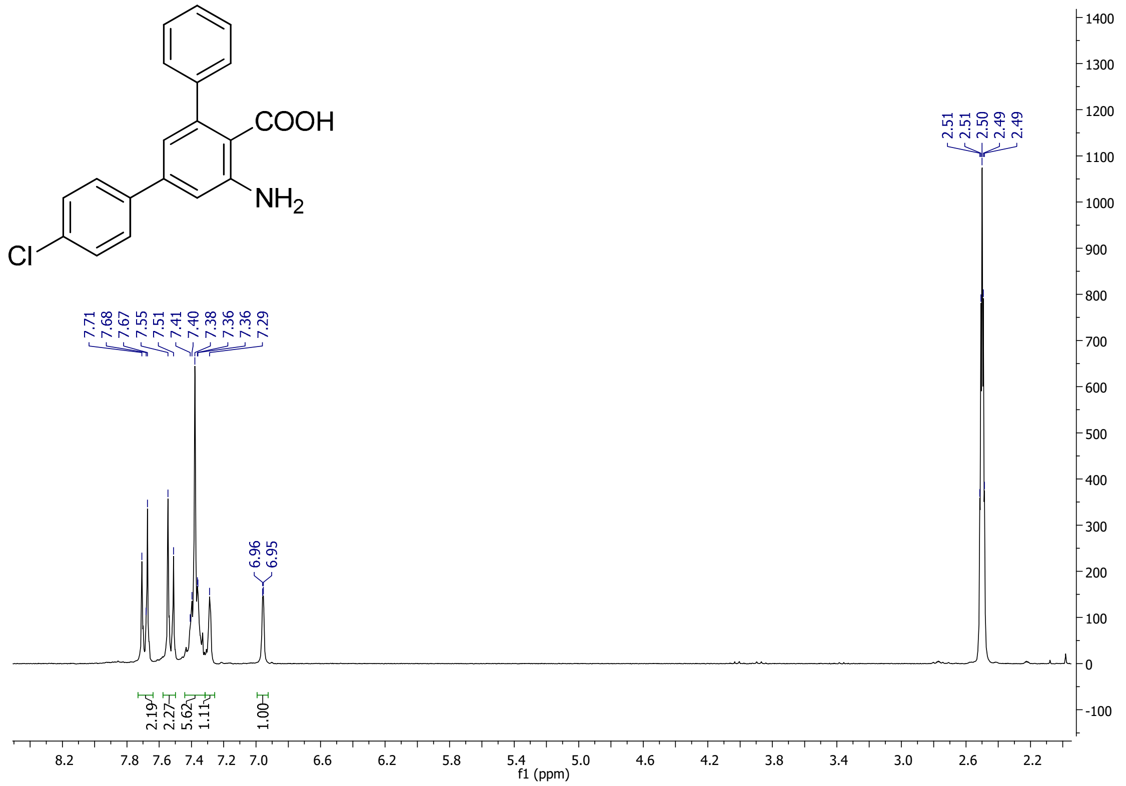

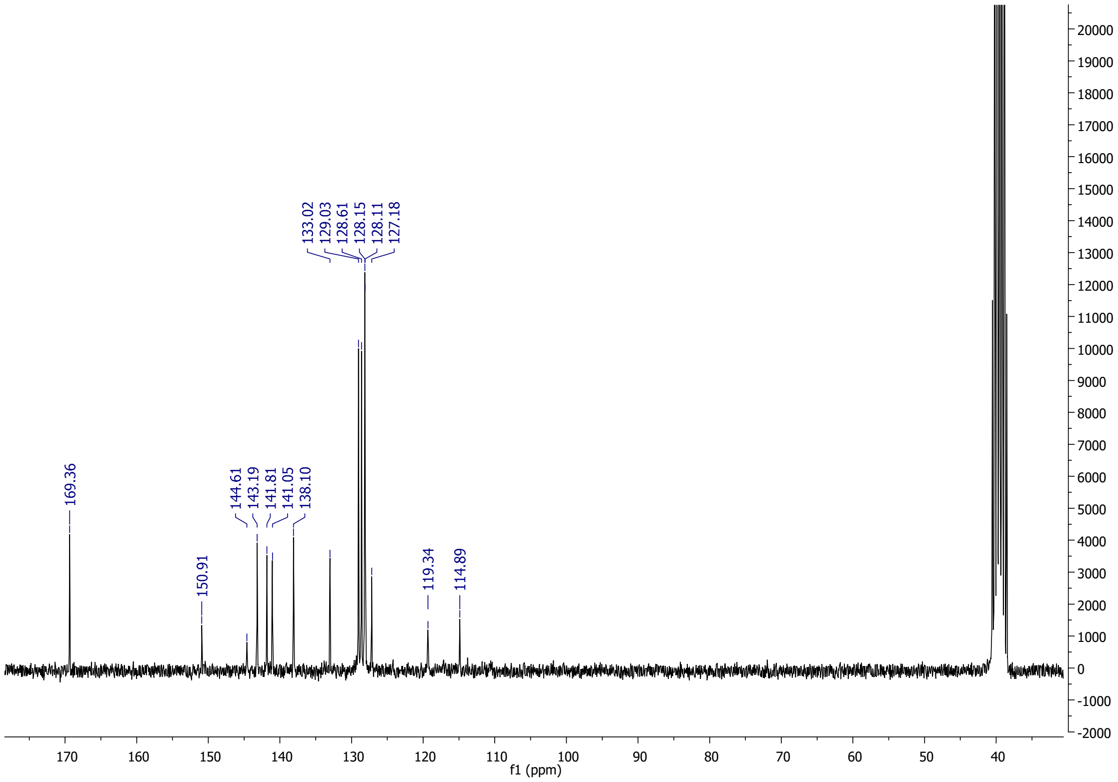

6-Amino-2,4-di-(4-chlorophenyl)benzoic acid (2d).

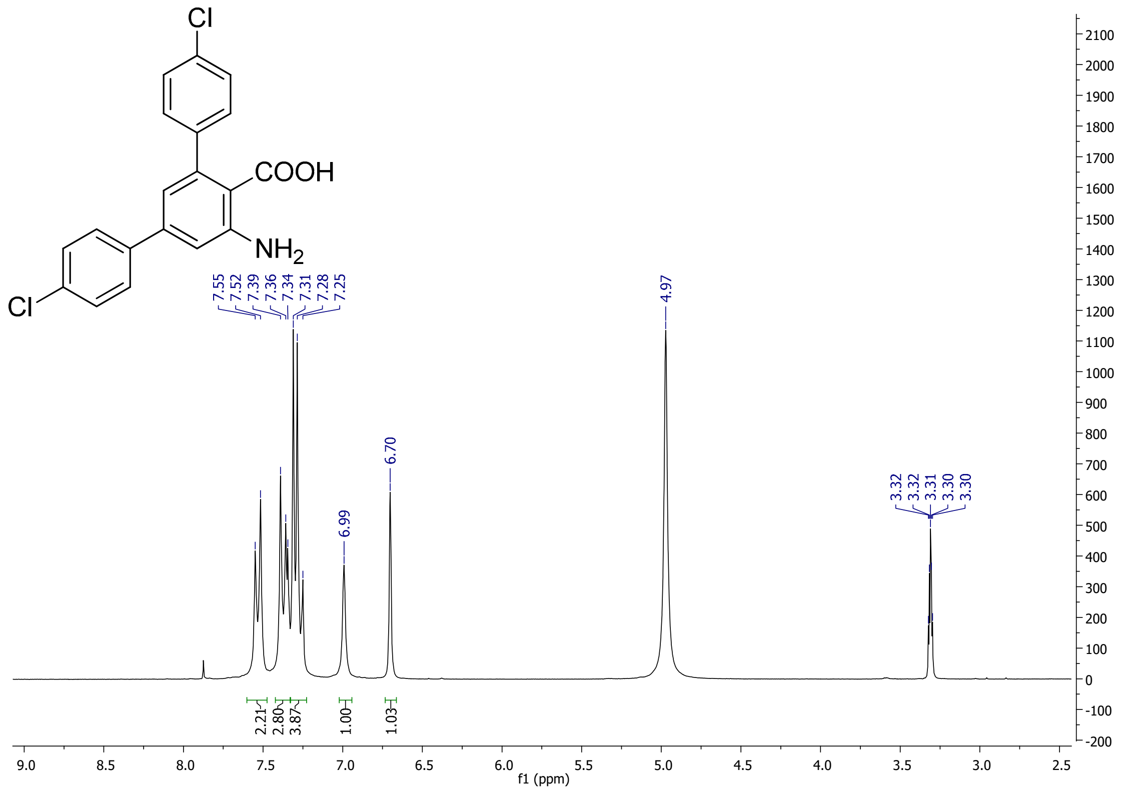

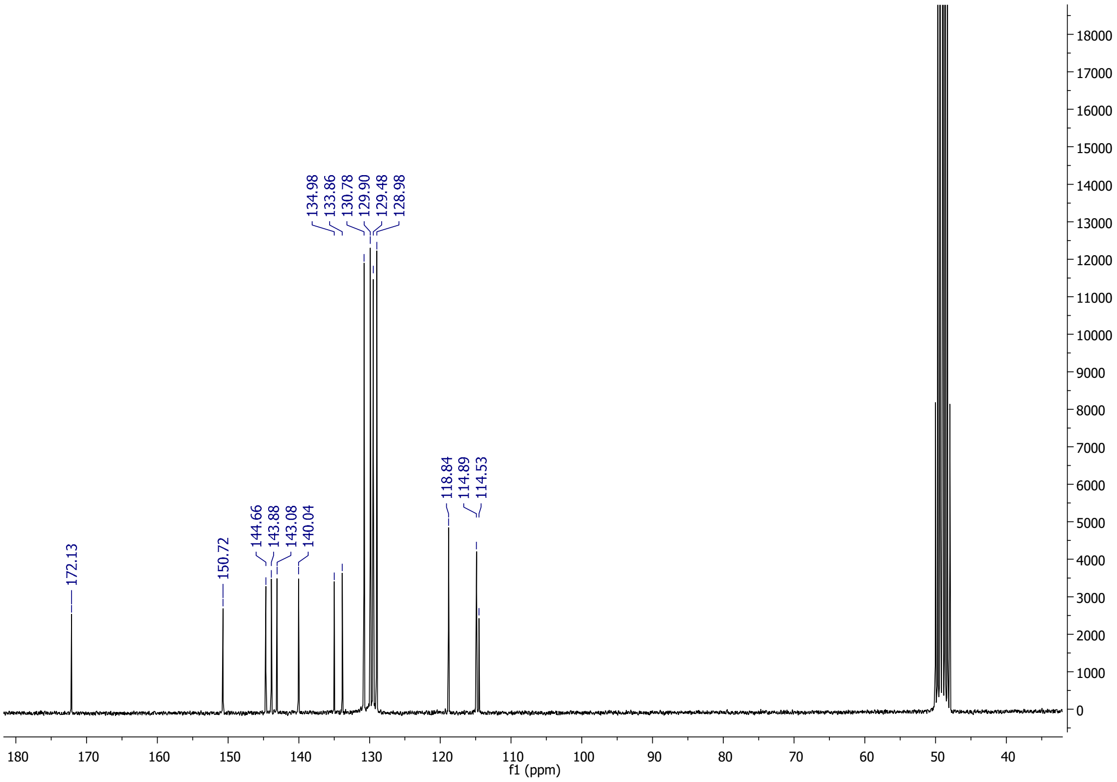

6-Amino-2-phenyl-4-(4-methoxyphenyl)benzoic acid (2e)

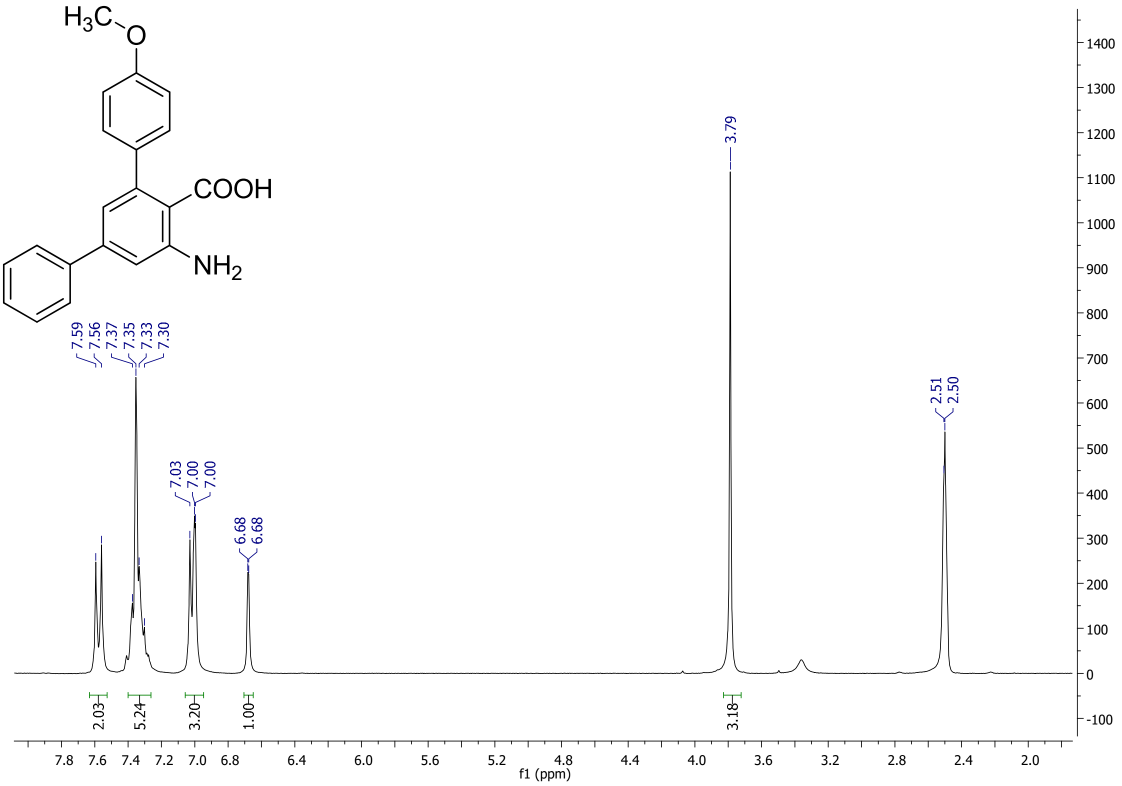

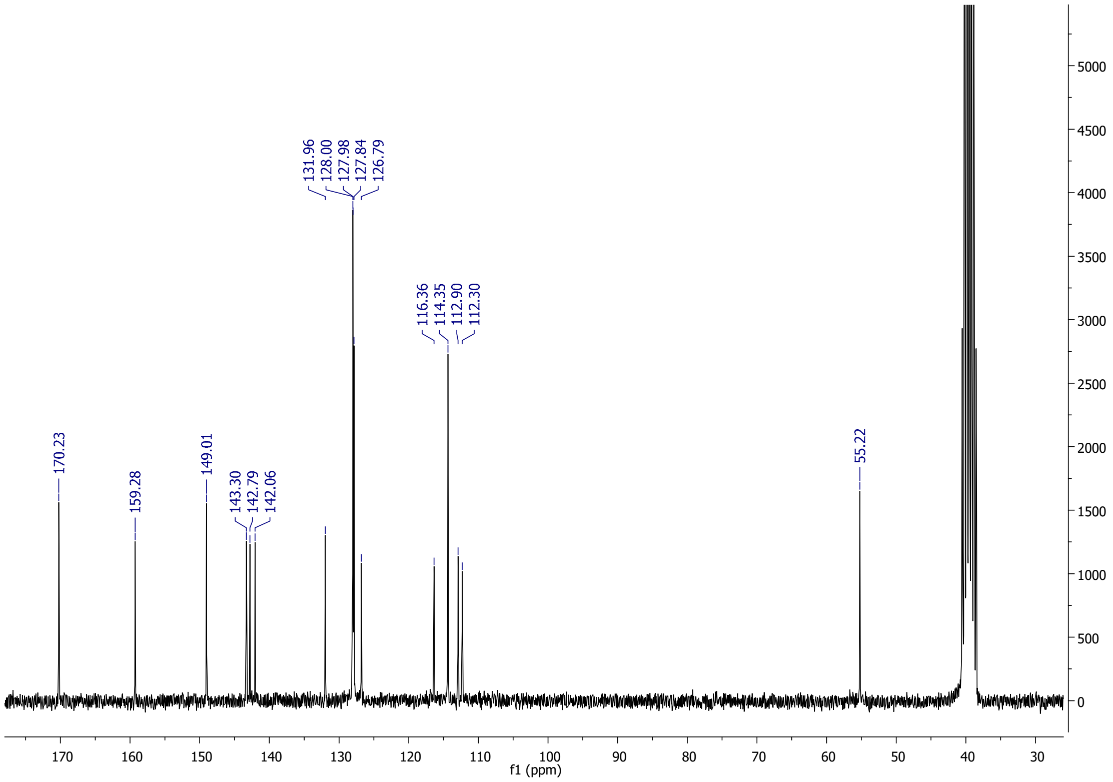

6-Amino-2,4-di-(4-bromophenyl) benzoic acid (2f)

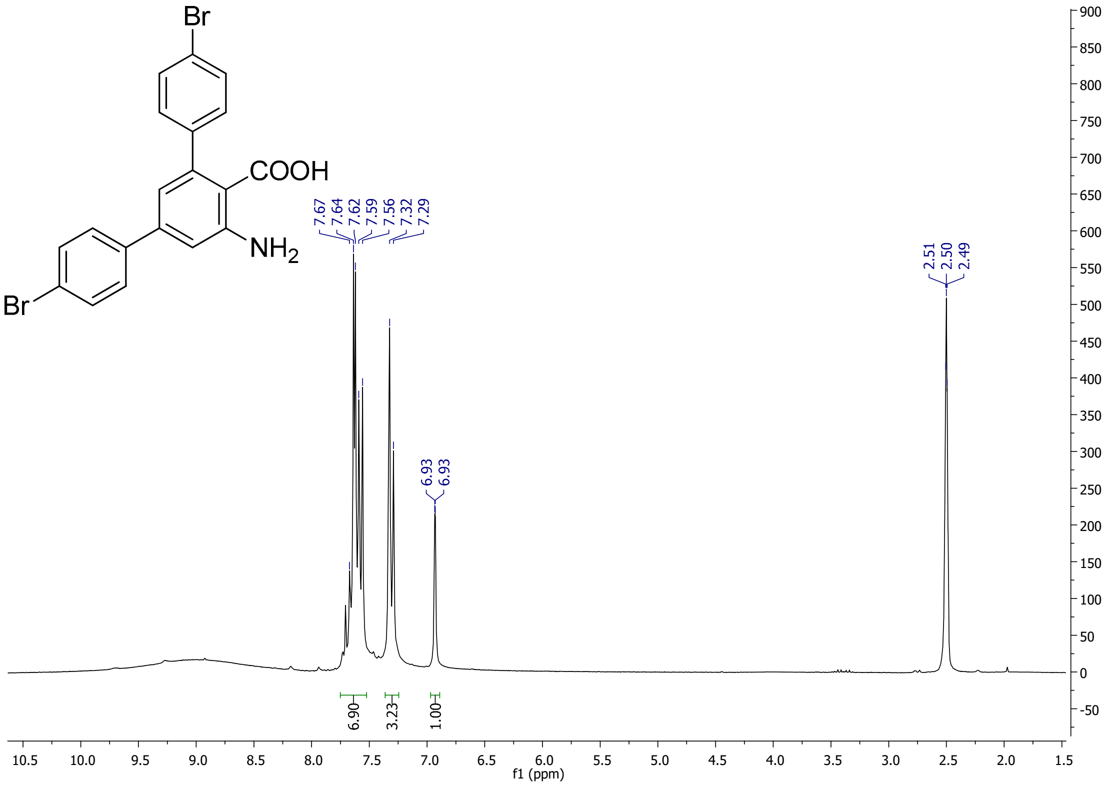

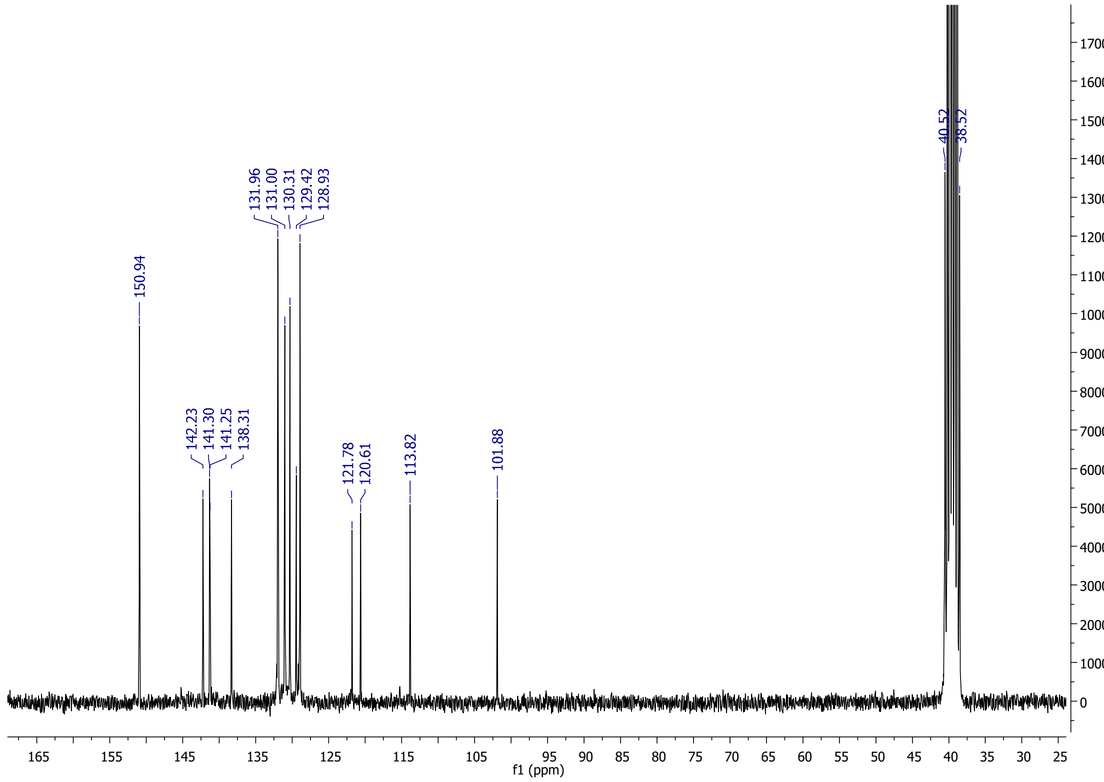

6-Phenylamino-2,4-diphenyl-benzoic acid (2g)

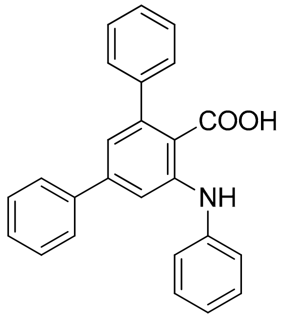

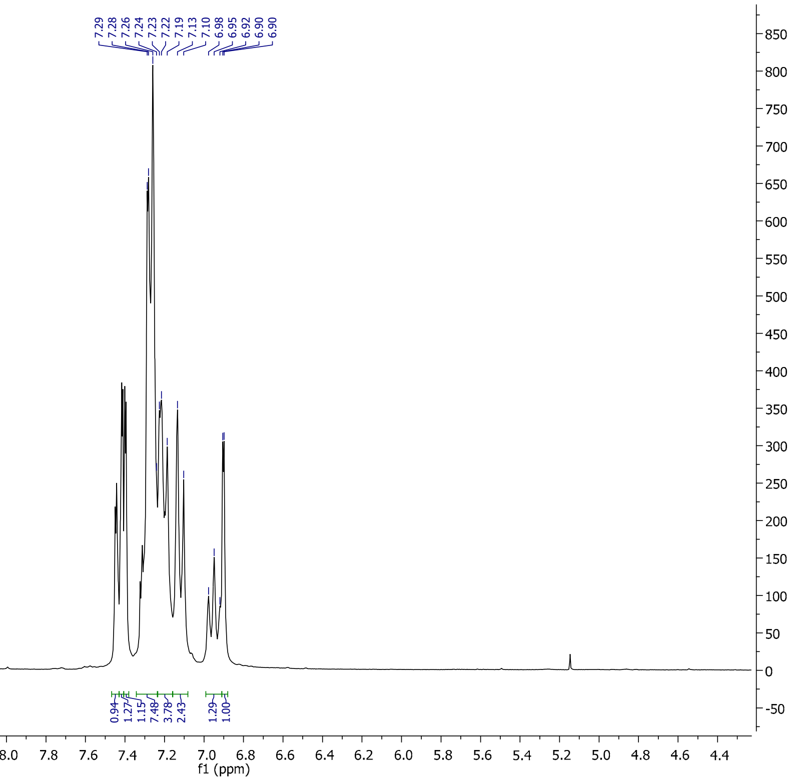

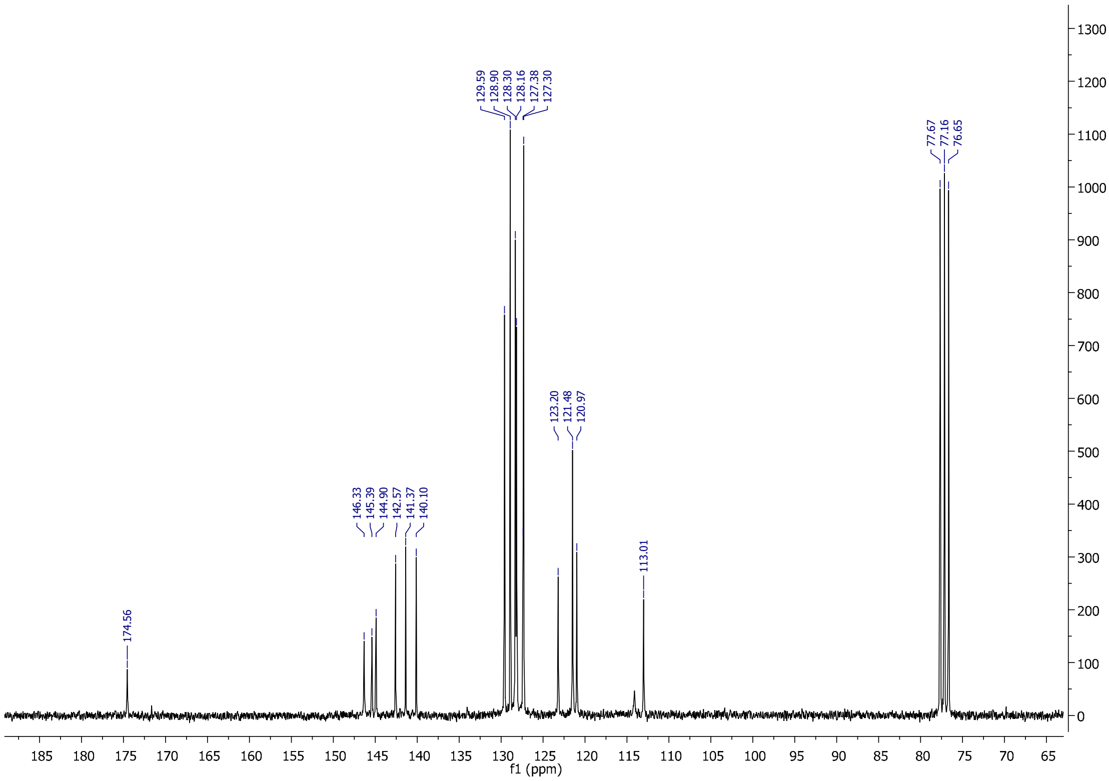

5'-Amino-*m*-terphenyl (3a)

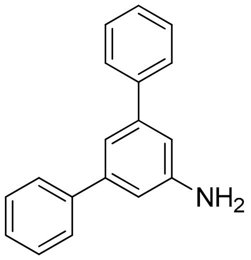

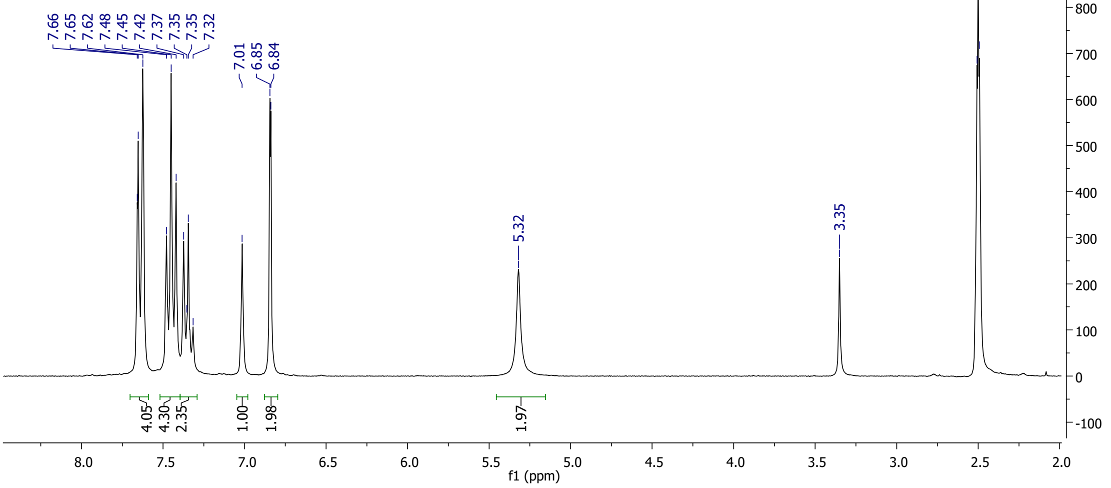

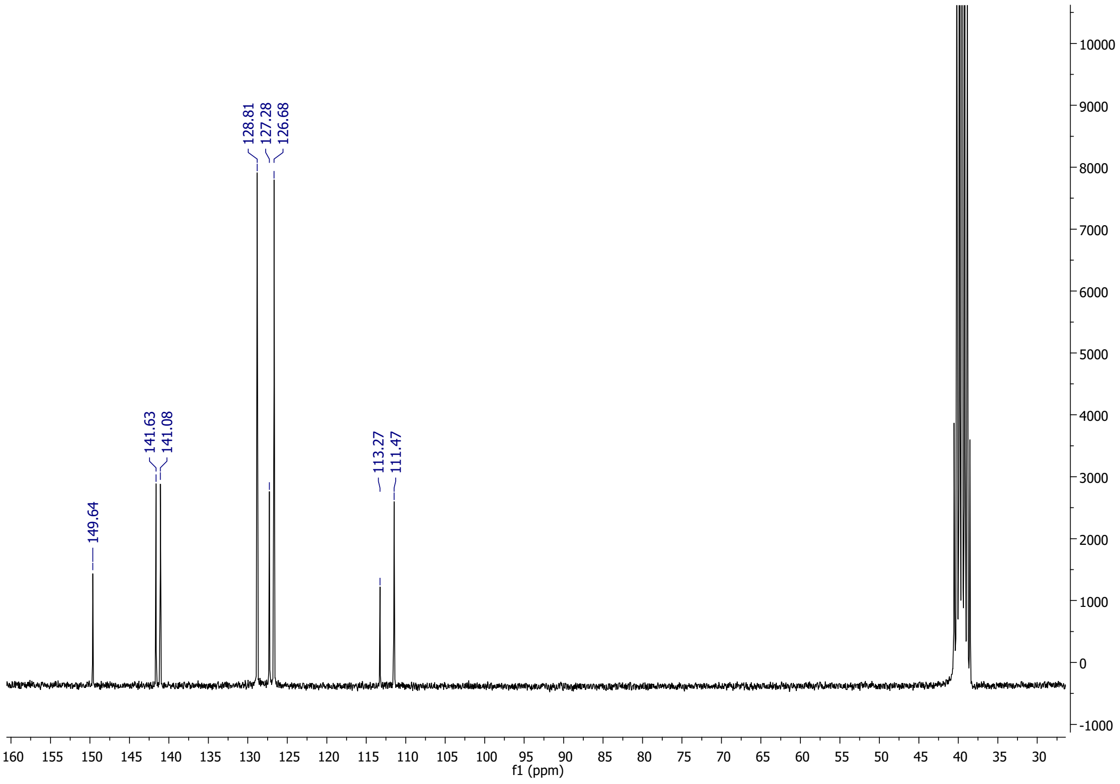

4-Methyl-5'-amino-*m*-terphenyl (3b)

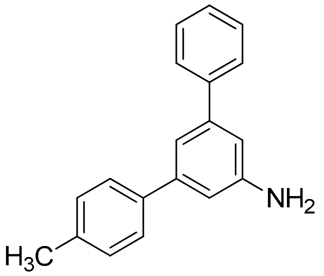

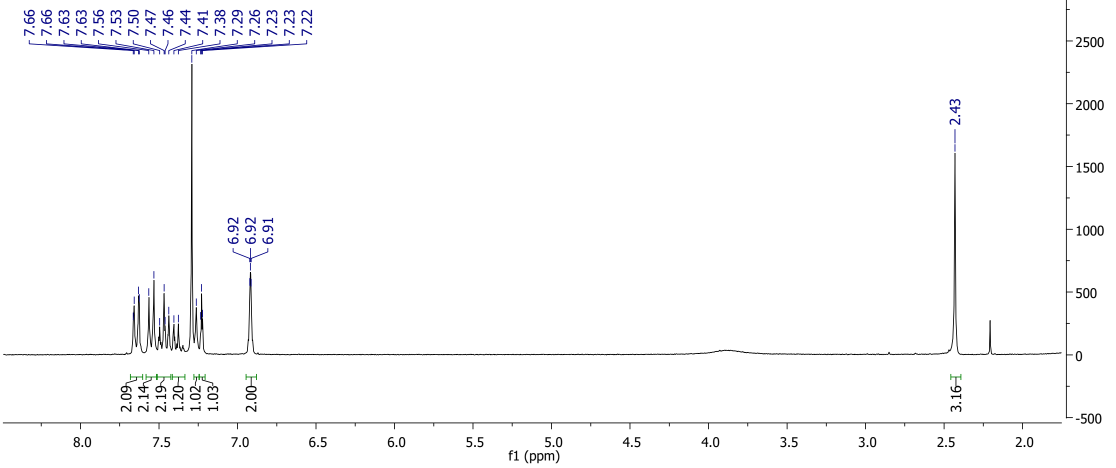

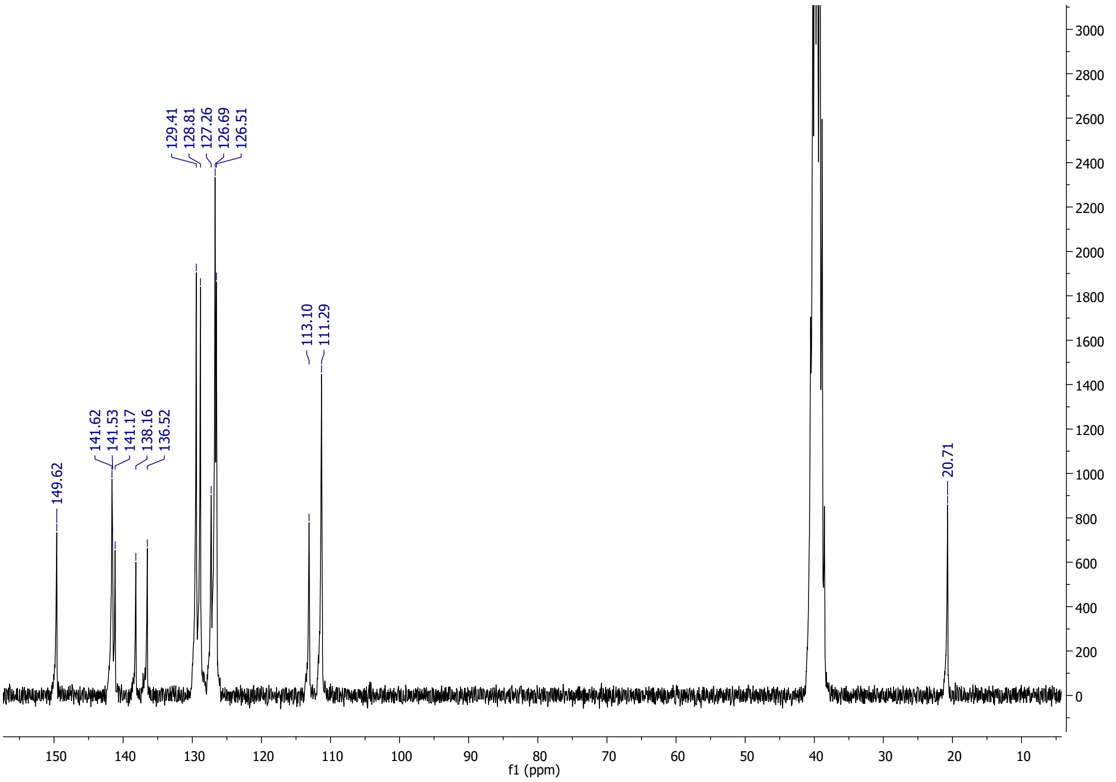

4-Chloro-5'-amino-*m*-terphenyl (3c)

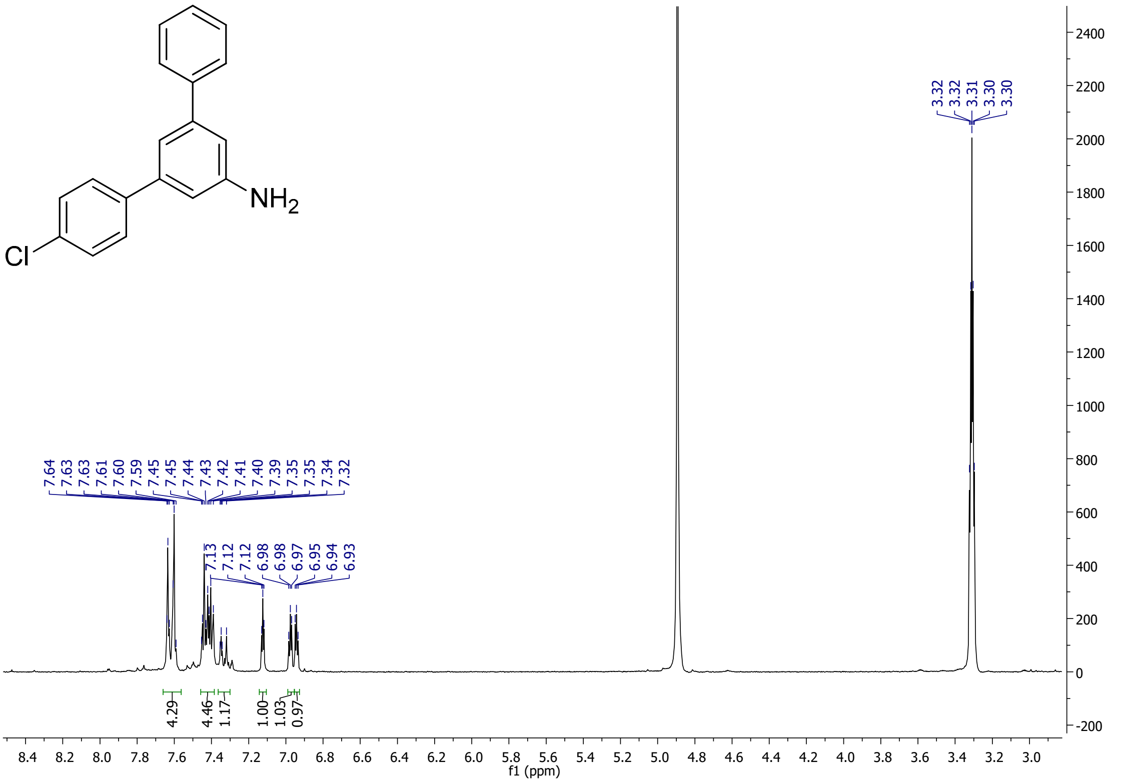

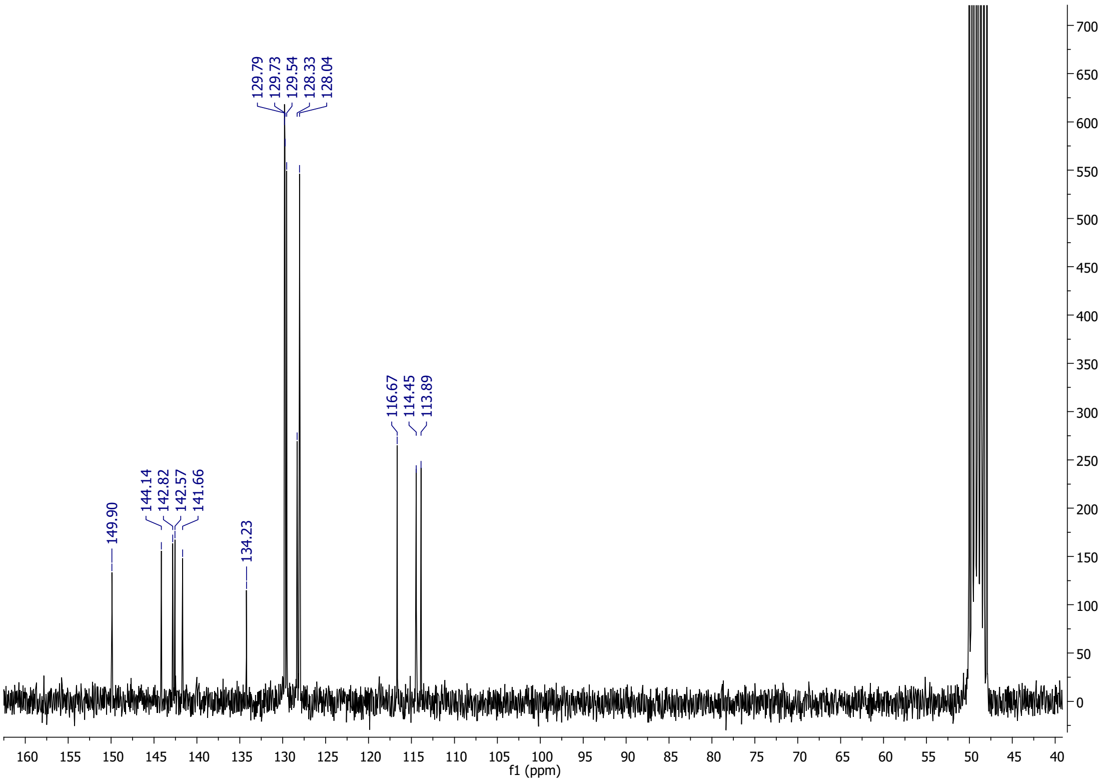

4,4''-Dichloro-5'-amino-*m*-terphenyl (3d)

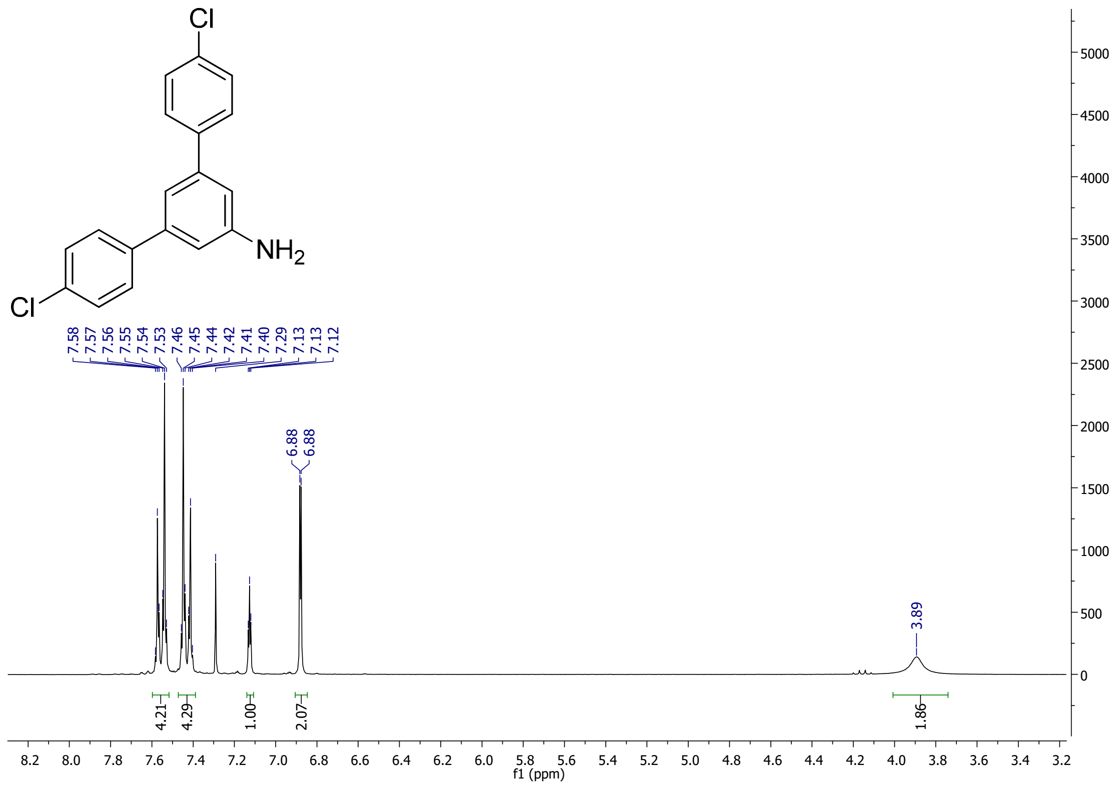

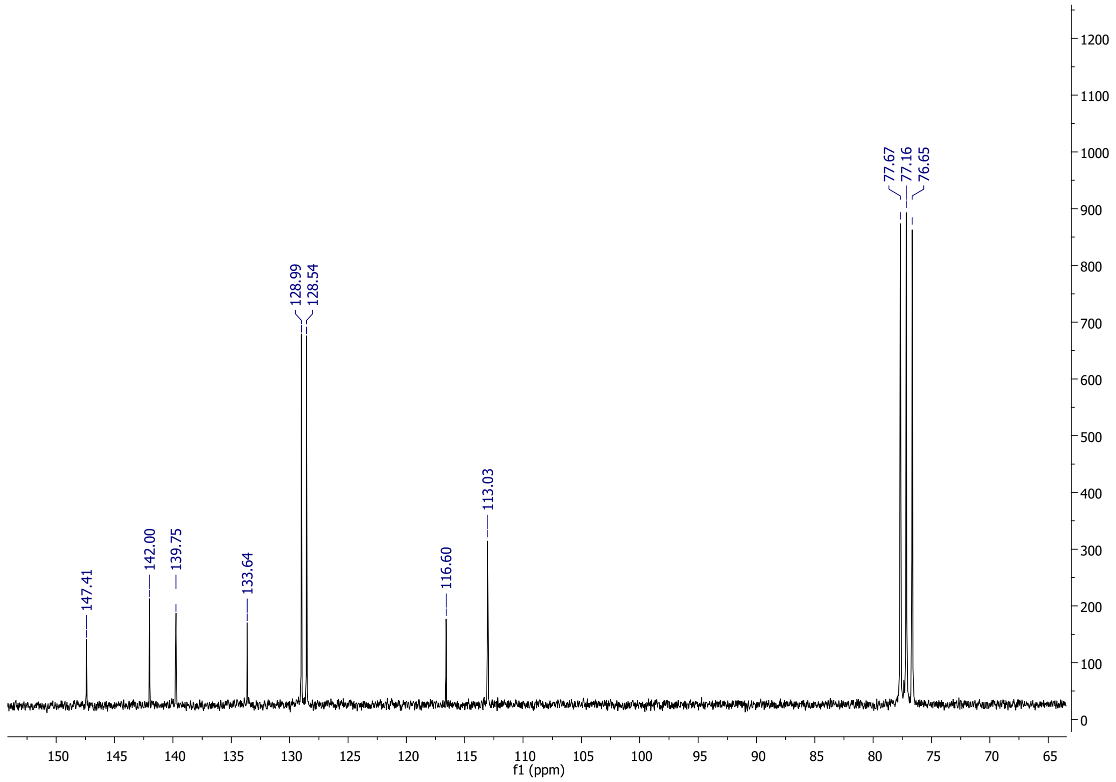

4-Methoxy-5'-amino-*m*-terphenyl (3e)

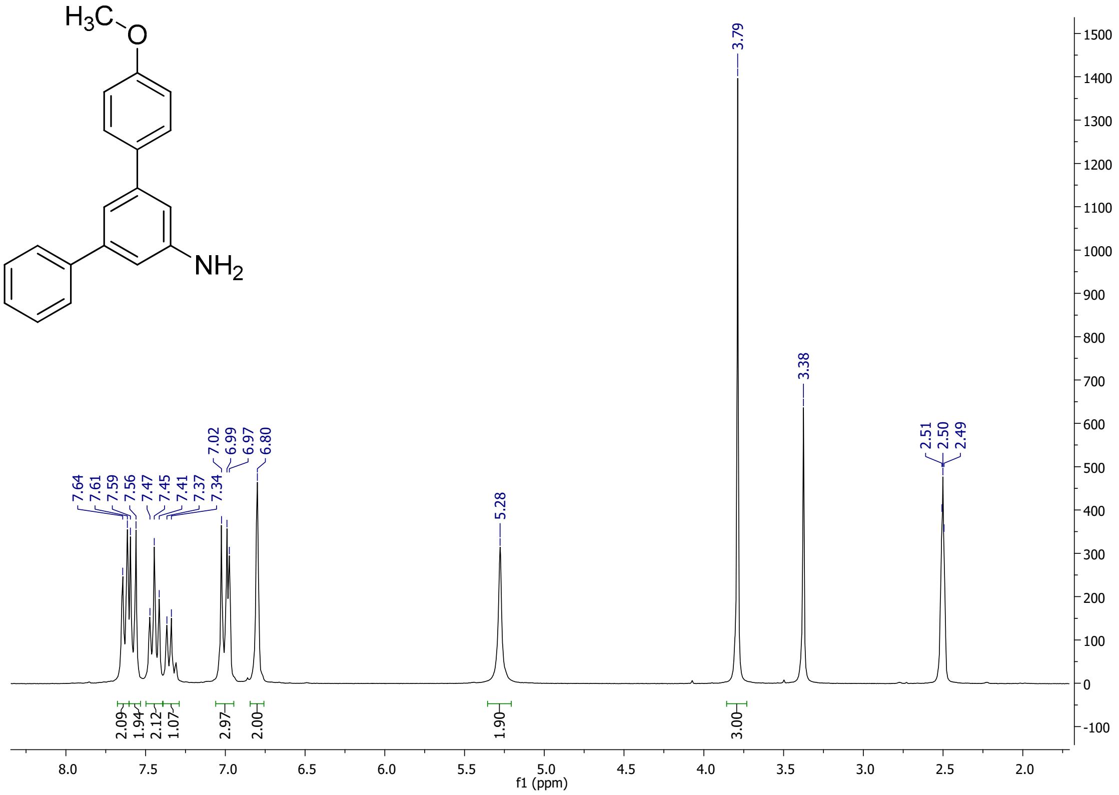

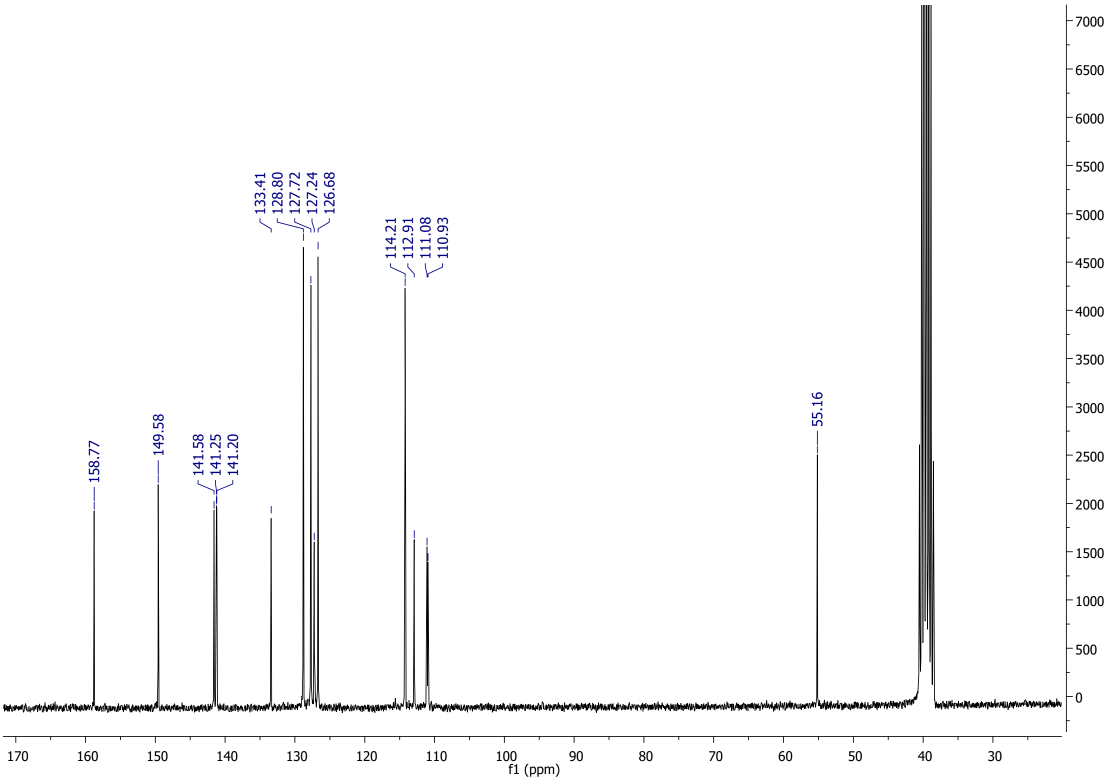

4,4''-Dibromo-5'-amino-*m*-terphenyl (3f)

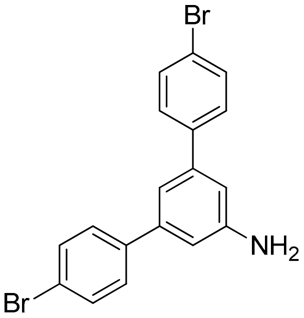

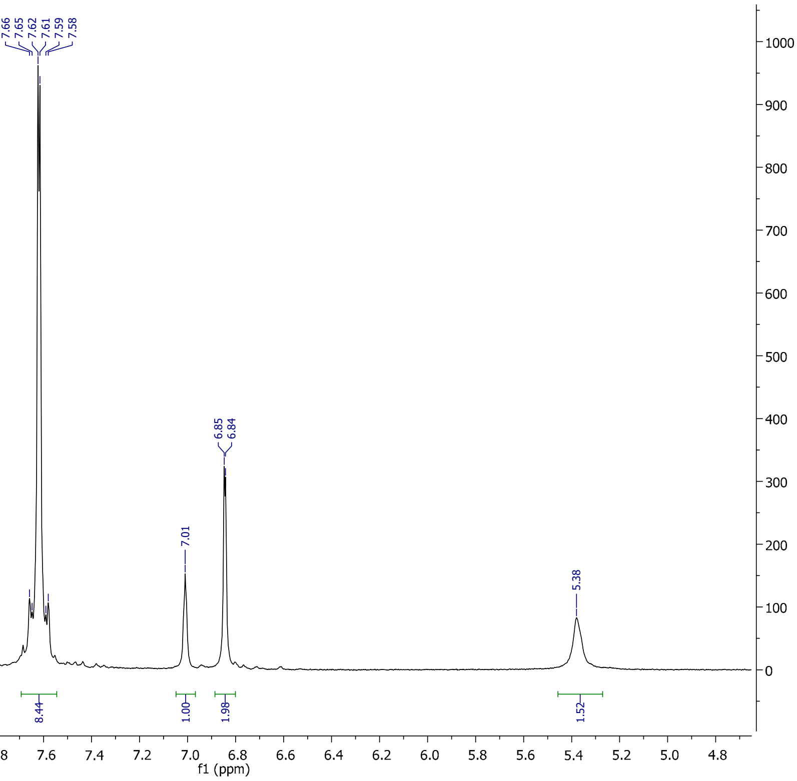

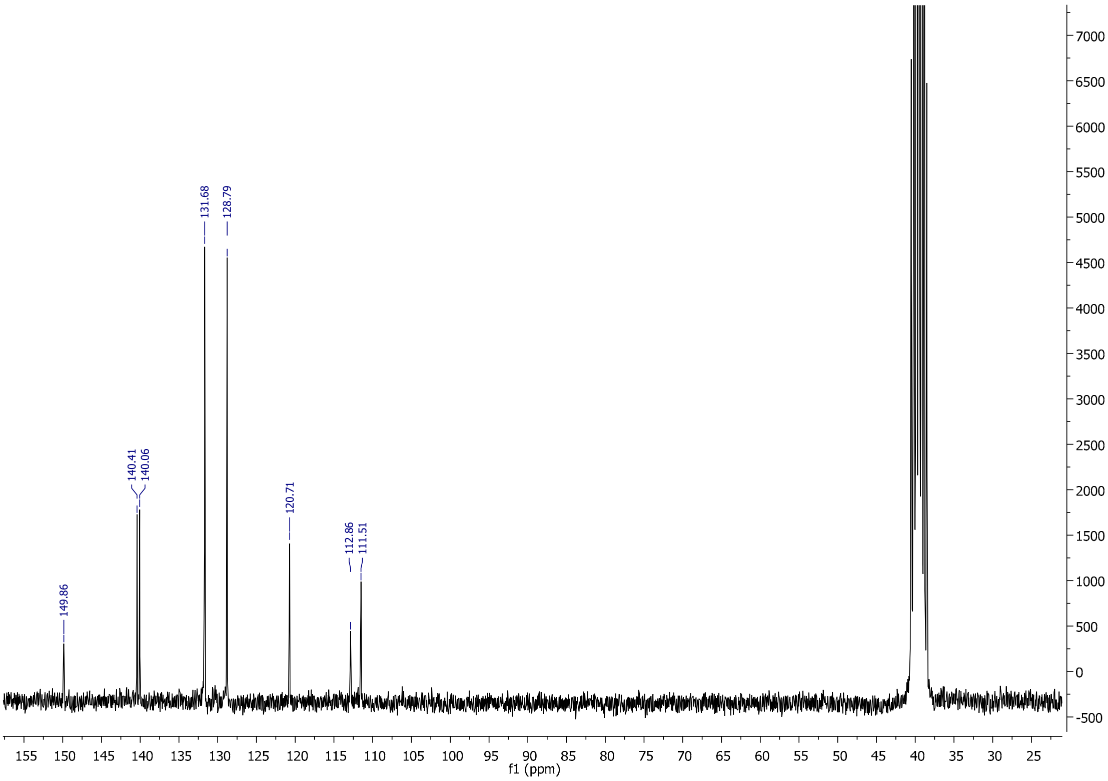

5'-Phenylamino-*m*-terphenyl (3g)

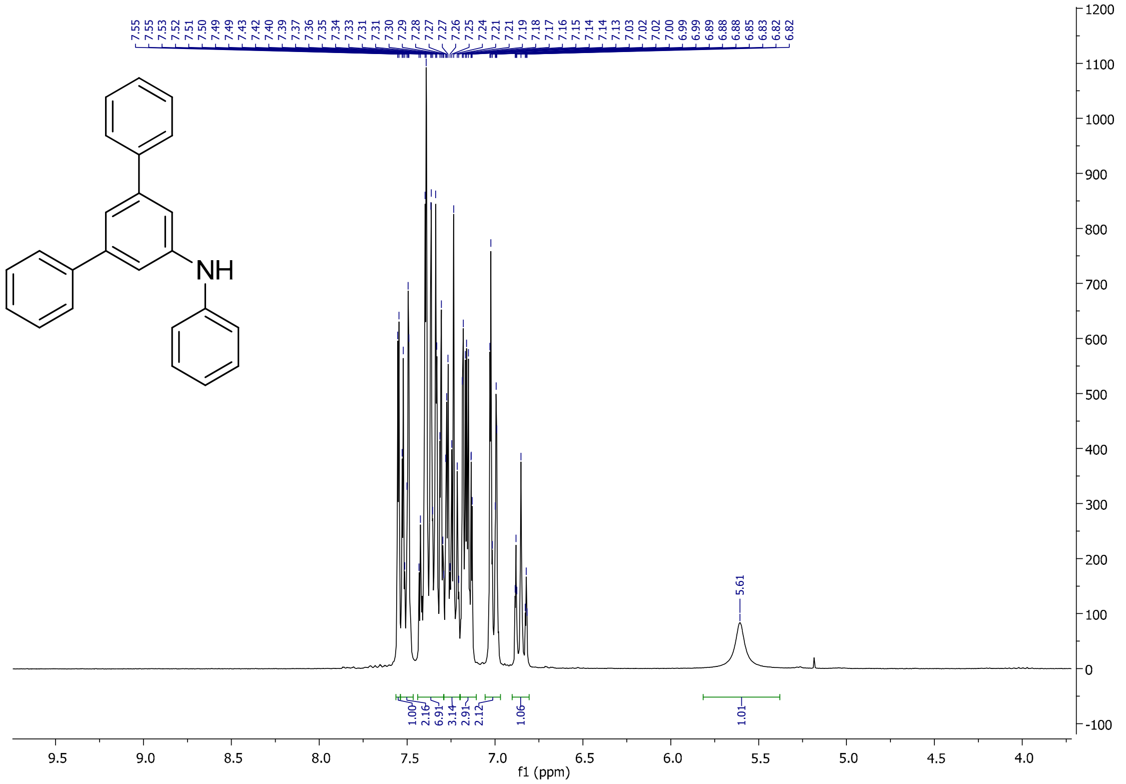

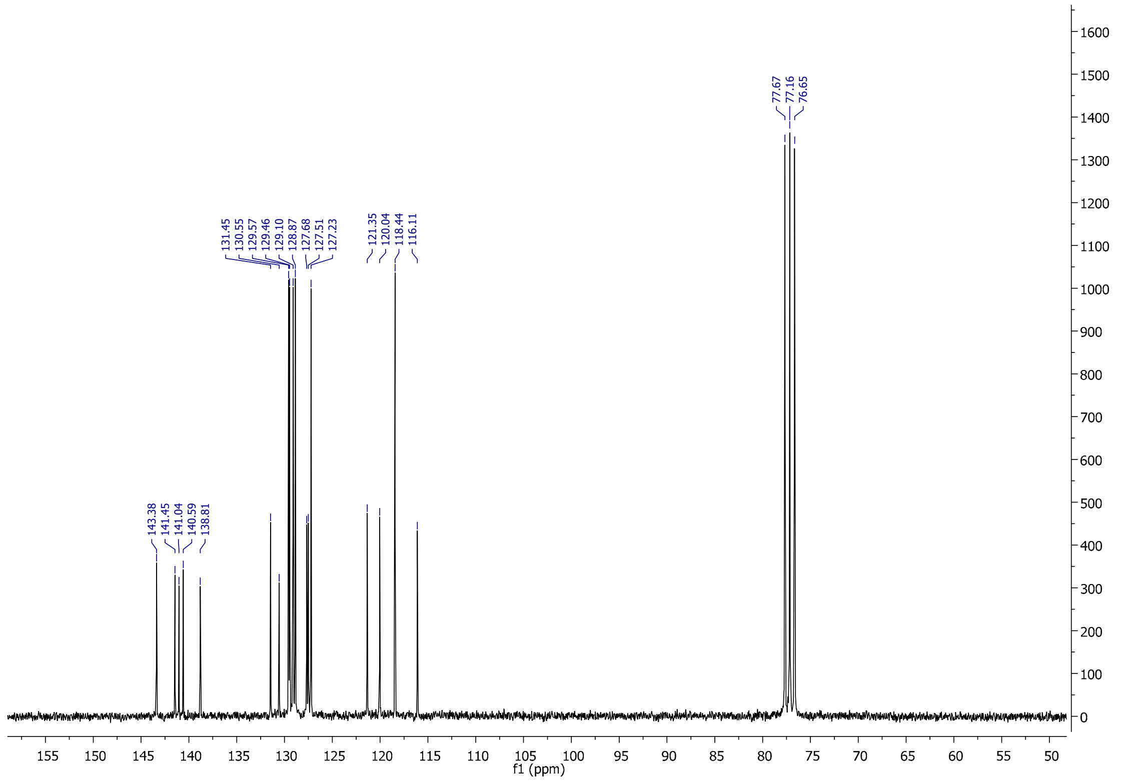

1-([*m*-Terphenyl]-5'-yl)-3-cyclopentylurea (4a)

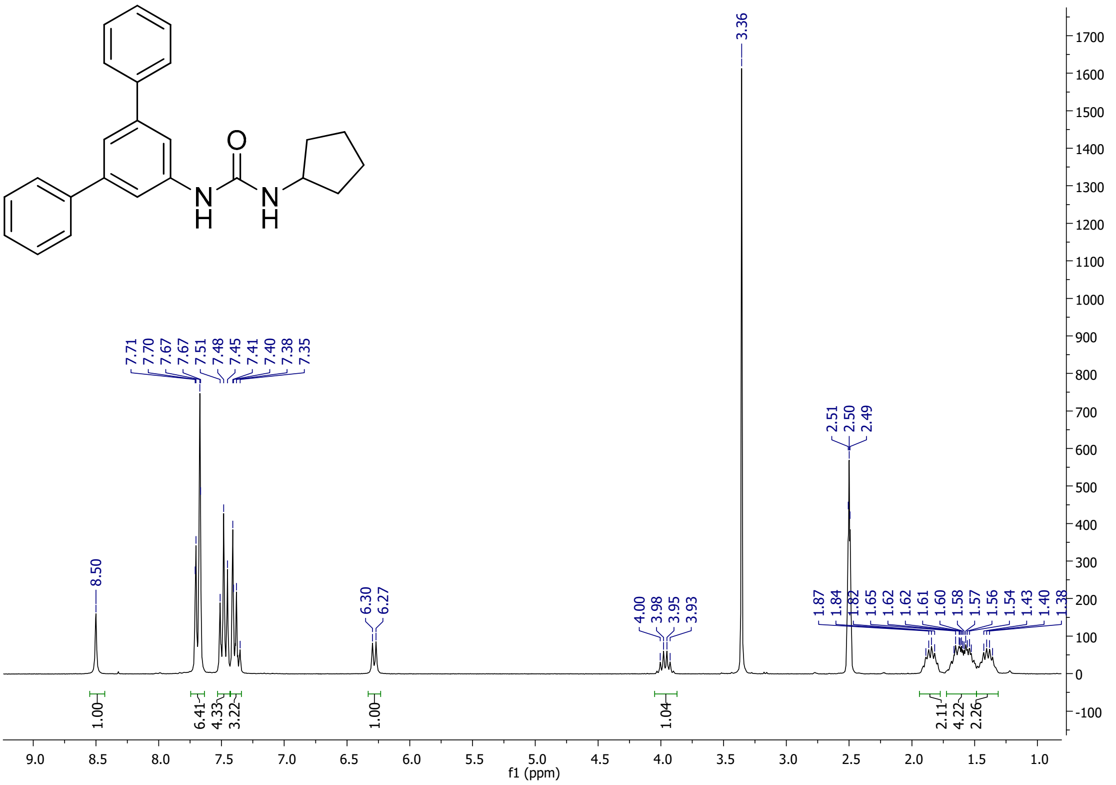

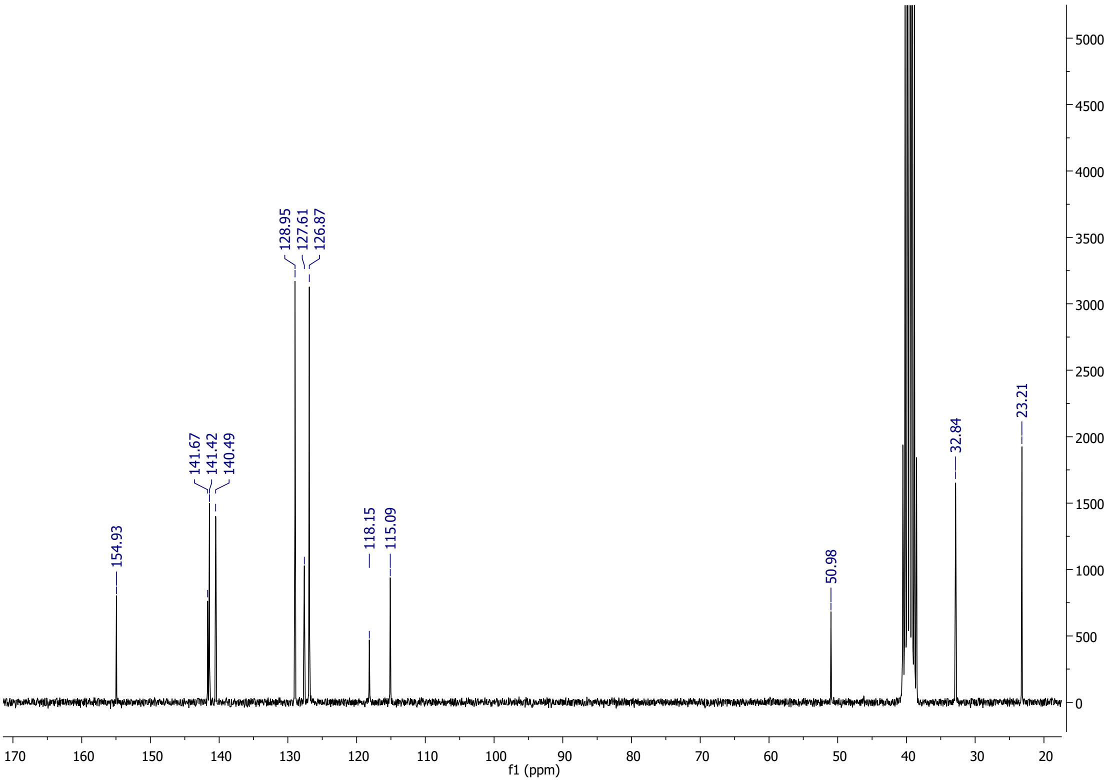

1-([4-Methyl-*m*-terphenyl]-5'-yl)-3-cyclopentylurea (4b)

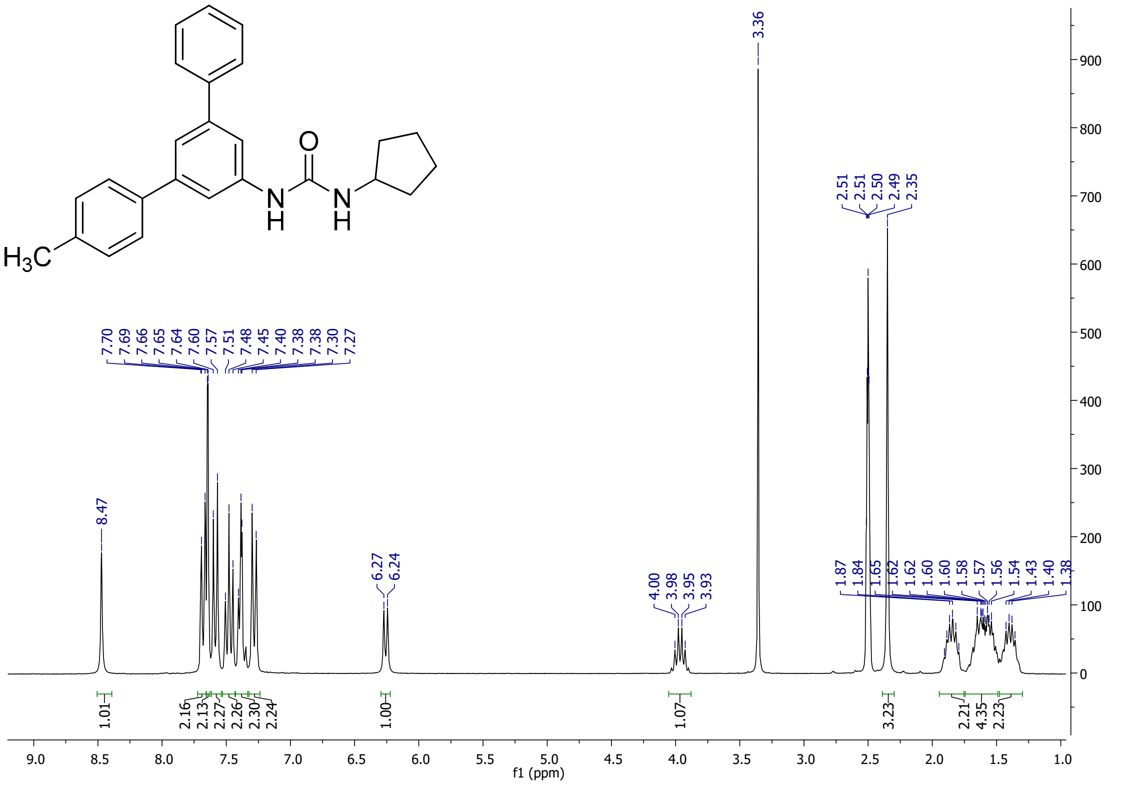

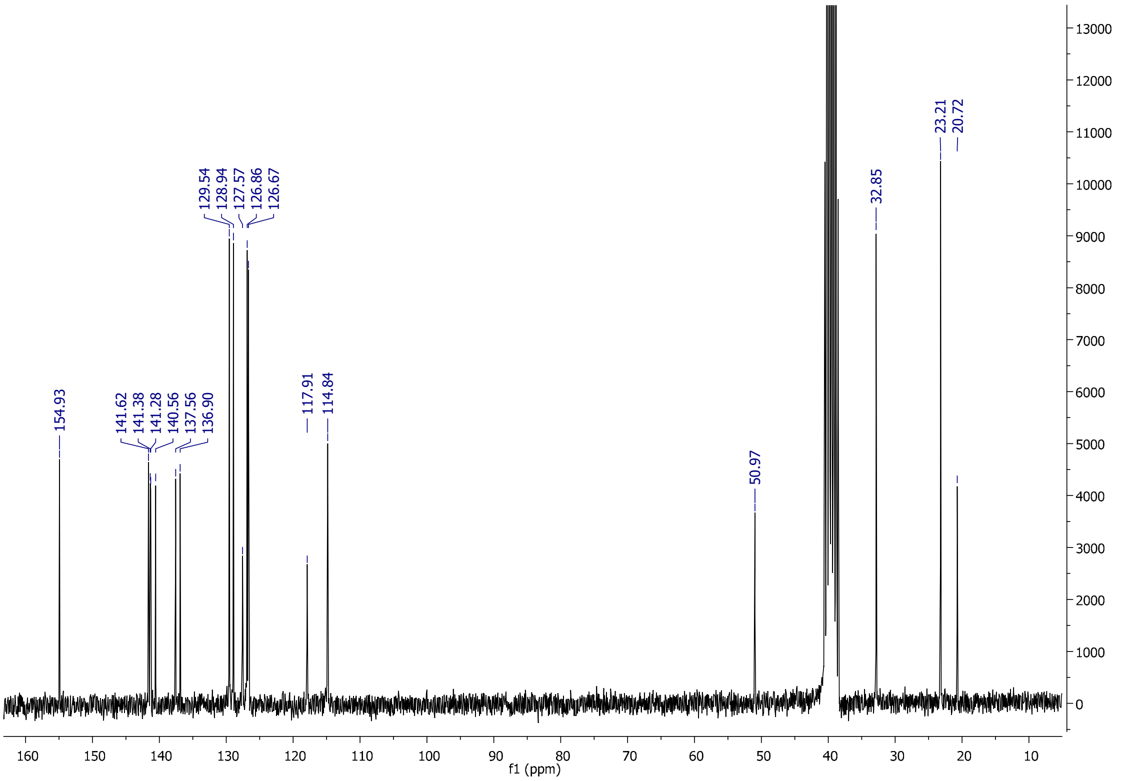

1-([4-Chloro-*m*-terphenyl]-5'-yl)-3-cyclopentylurea (4c)

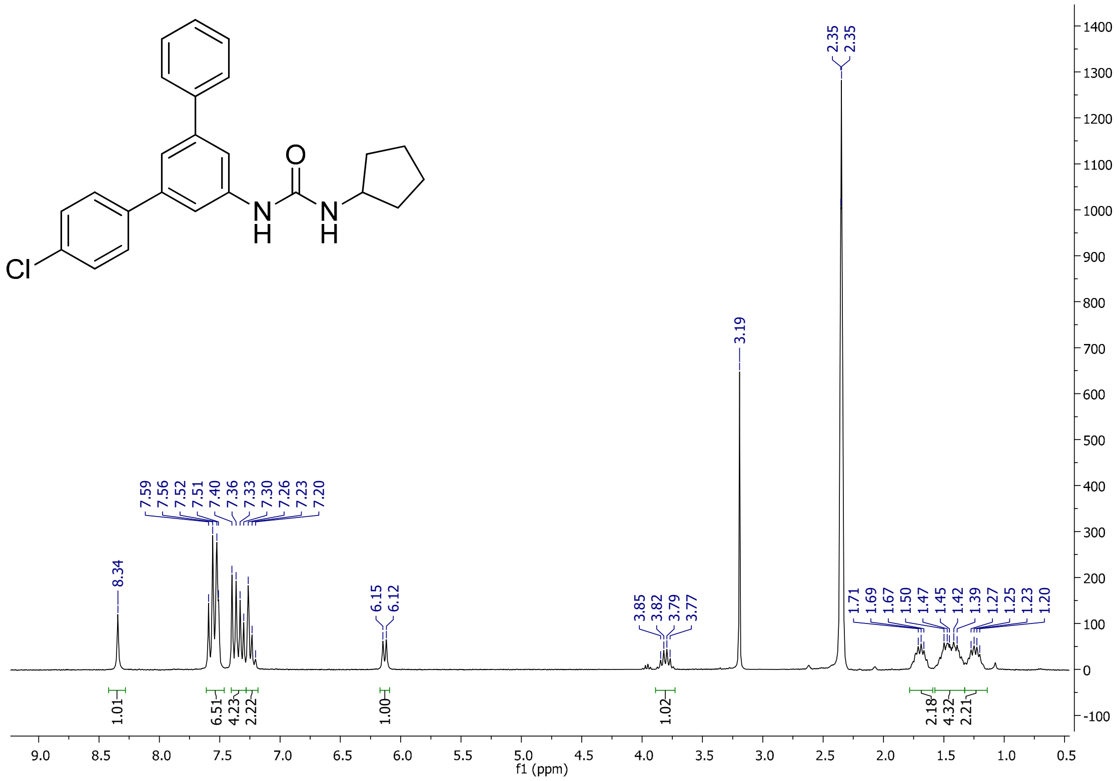

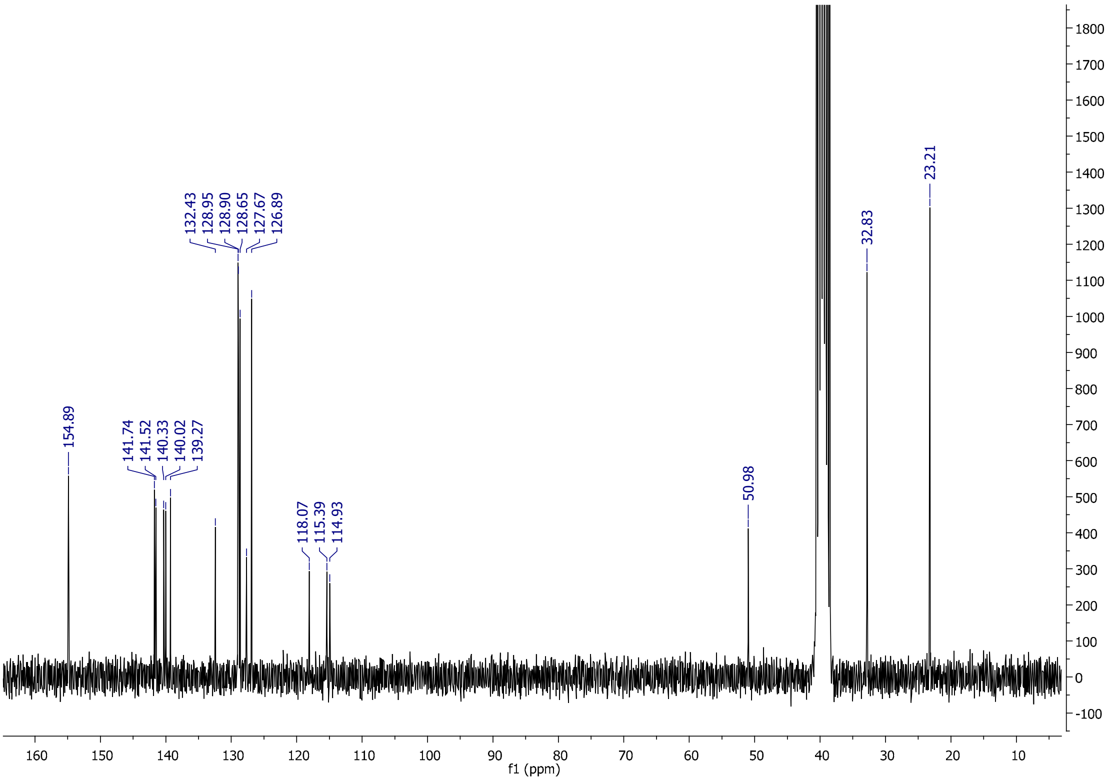

1-([4,4''-Dichloro-*m*-terphenyl]-5'-yl)-3-cyclopentylurea (4d)

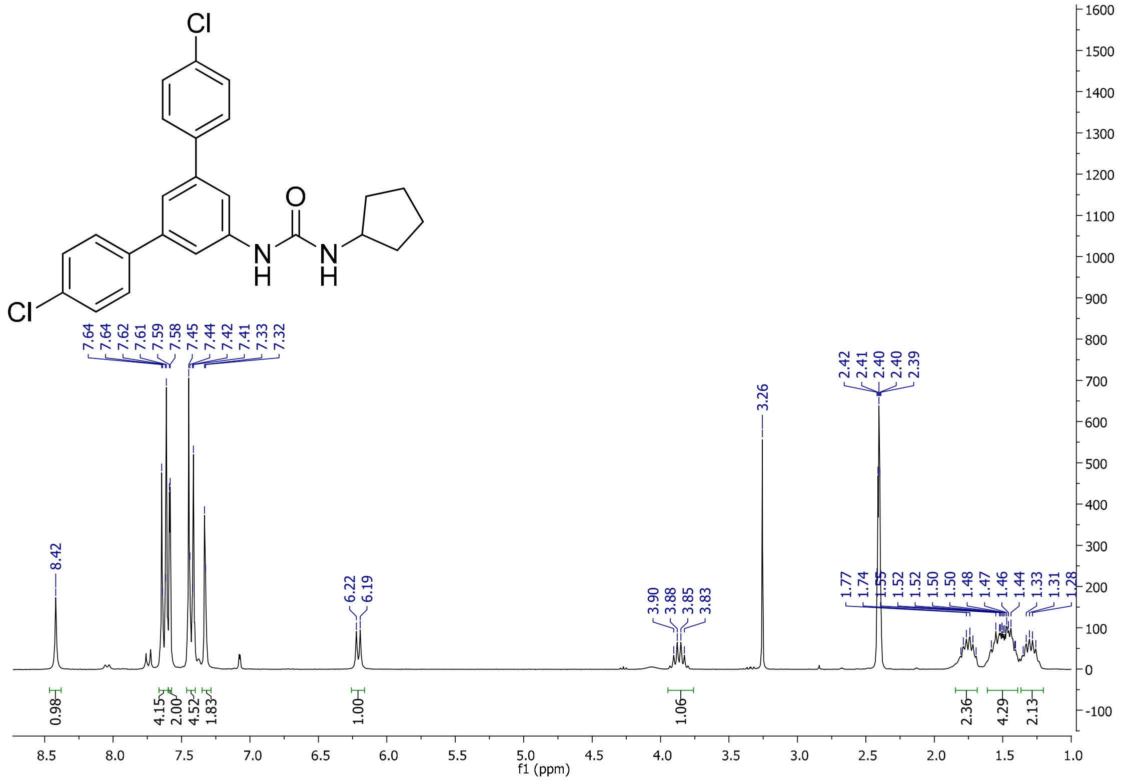

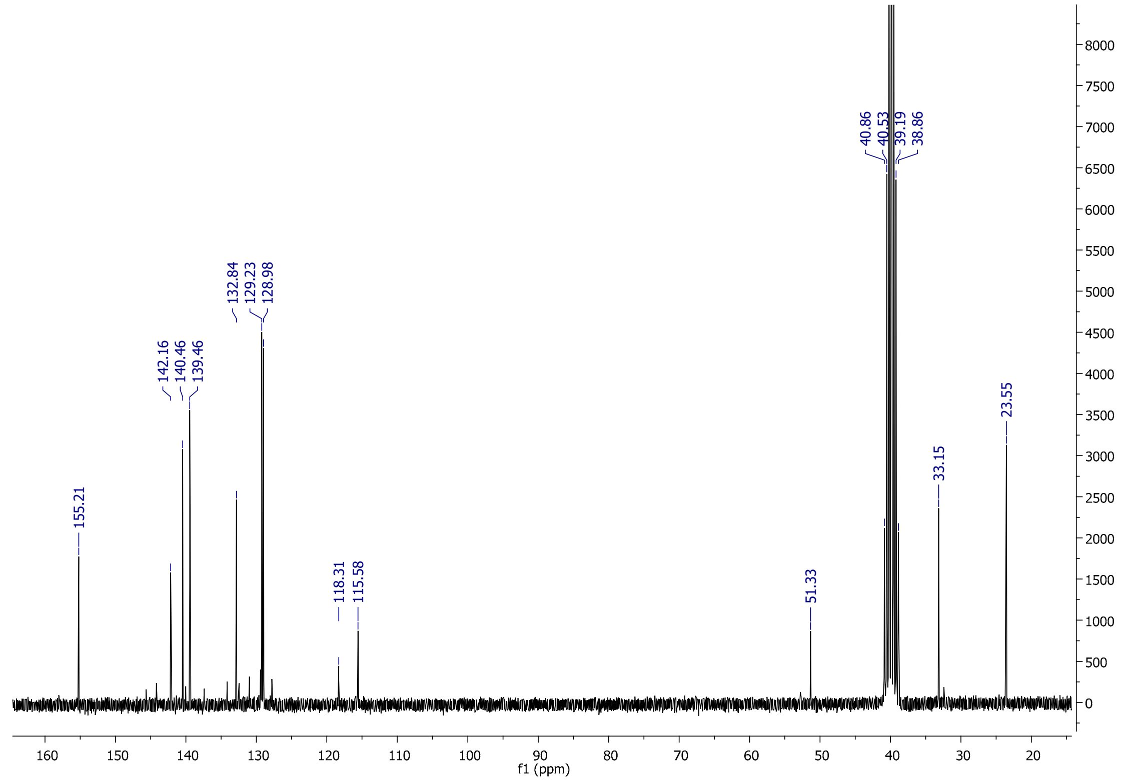

1-([4-Methoxy-*m*-terphenyl]-5'-yl)-3-cyclopentylurea (4e)

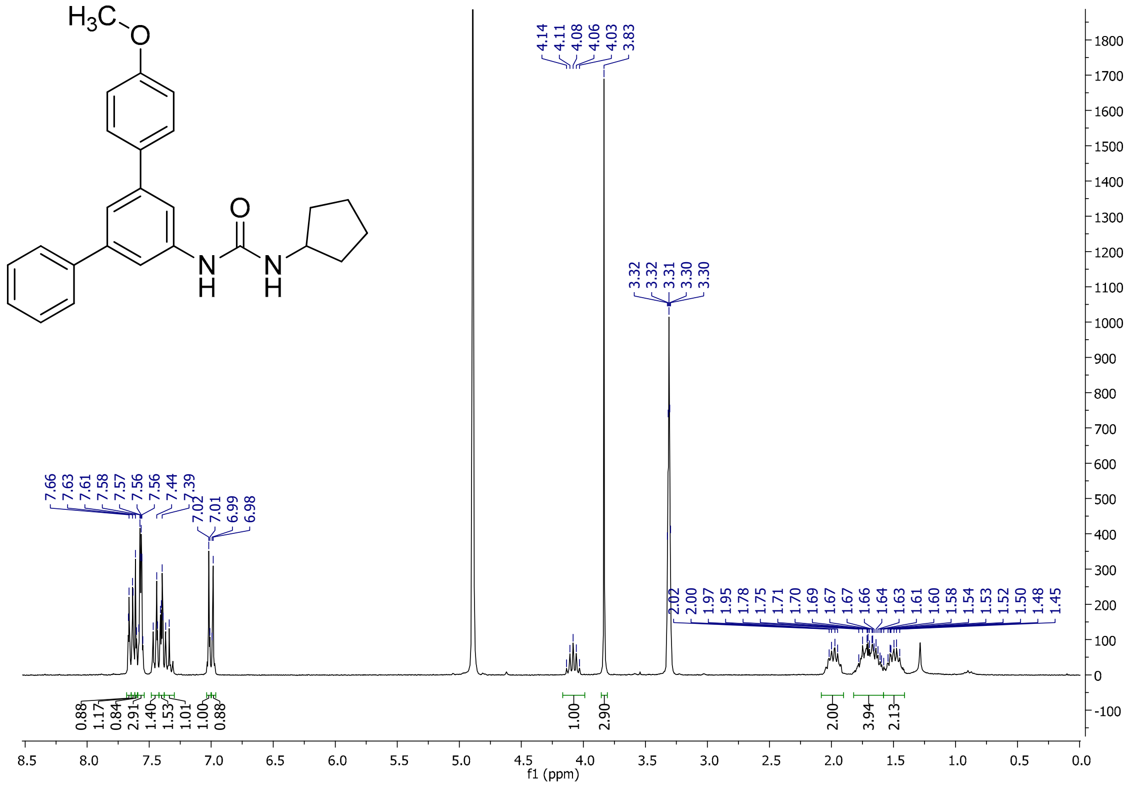

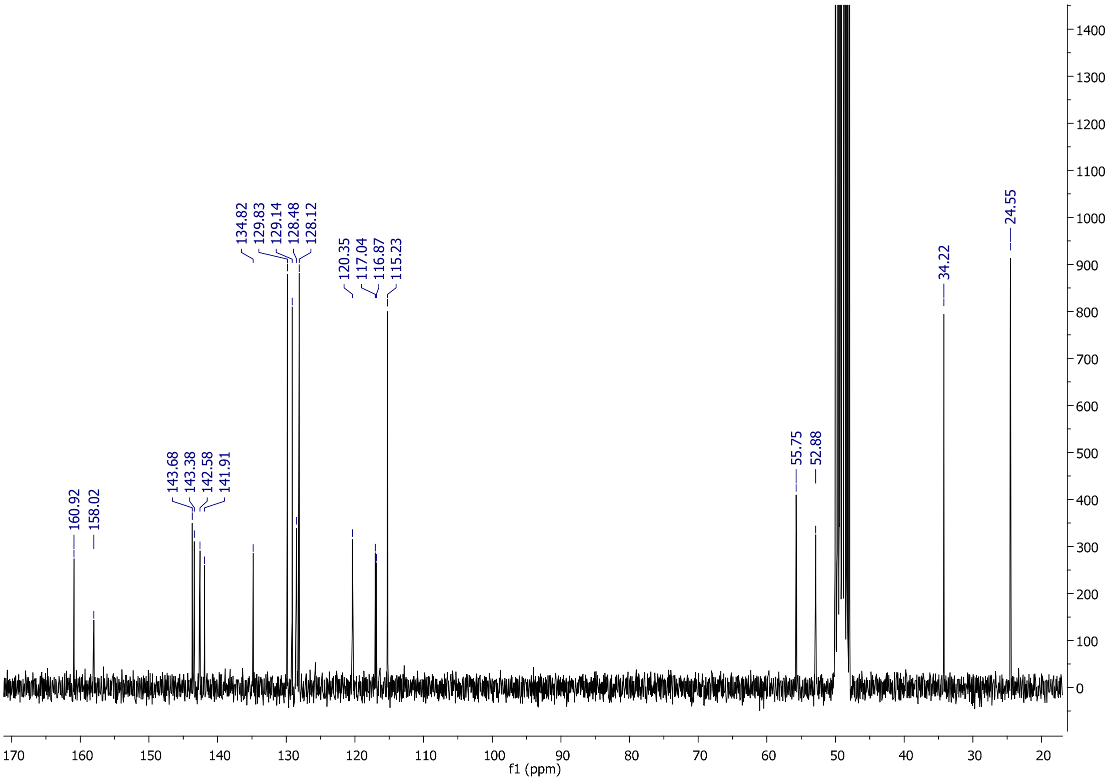

1-([4,4''-Dibromo-*m*-terphenyl]-5'-yl)-3-cyclopentylurea (4f)

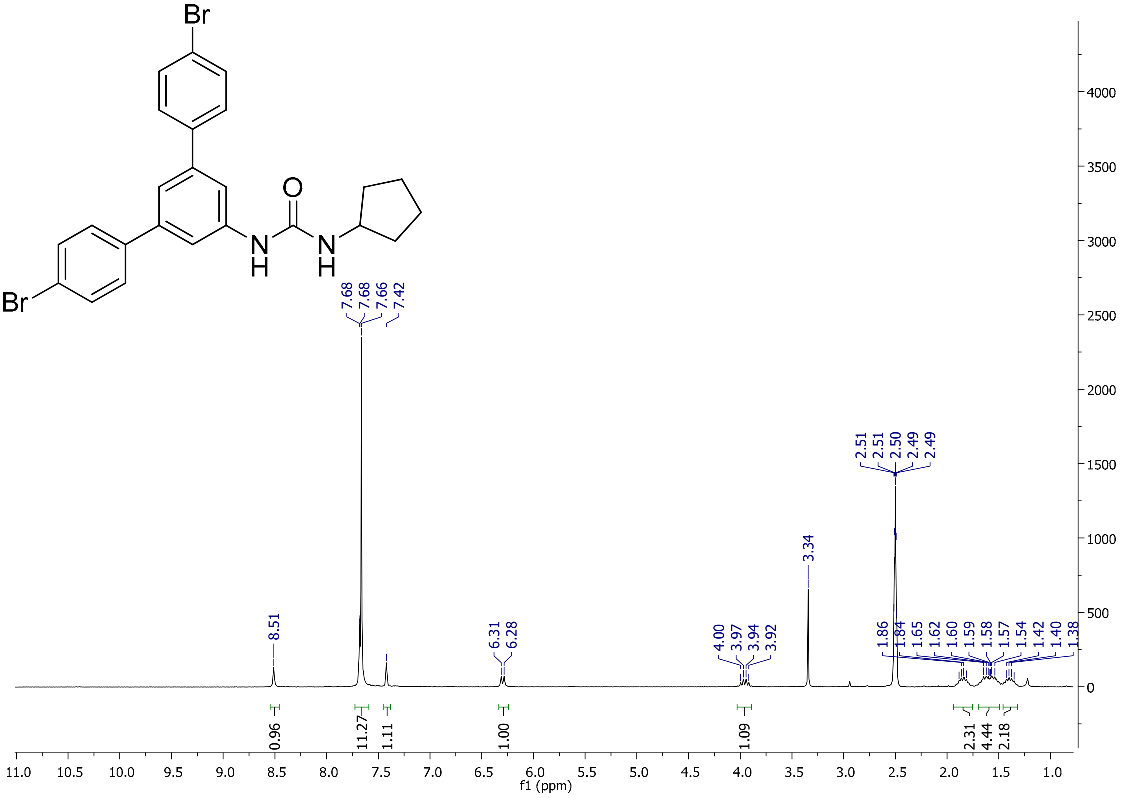

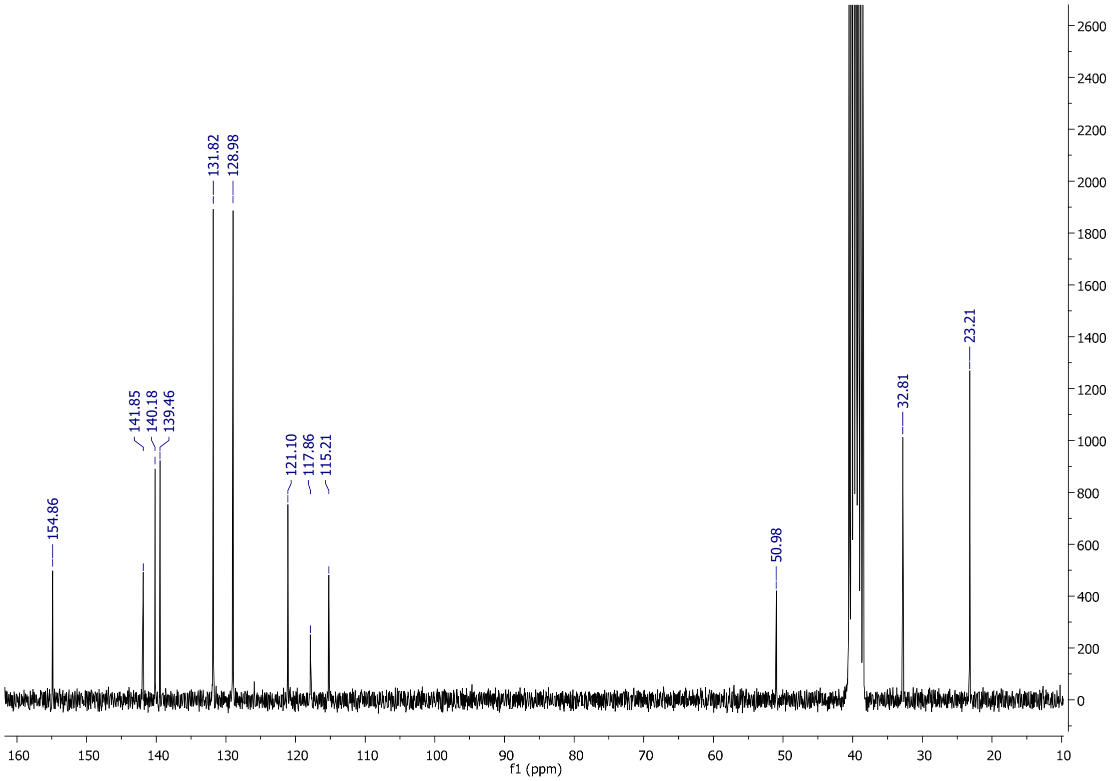

Ethyl 5'-(3-cyclohexylureido)-4-methoxy-*m*-terphenyl-4'-carboxylate (5)

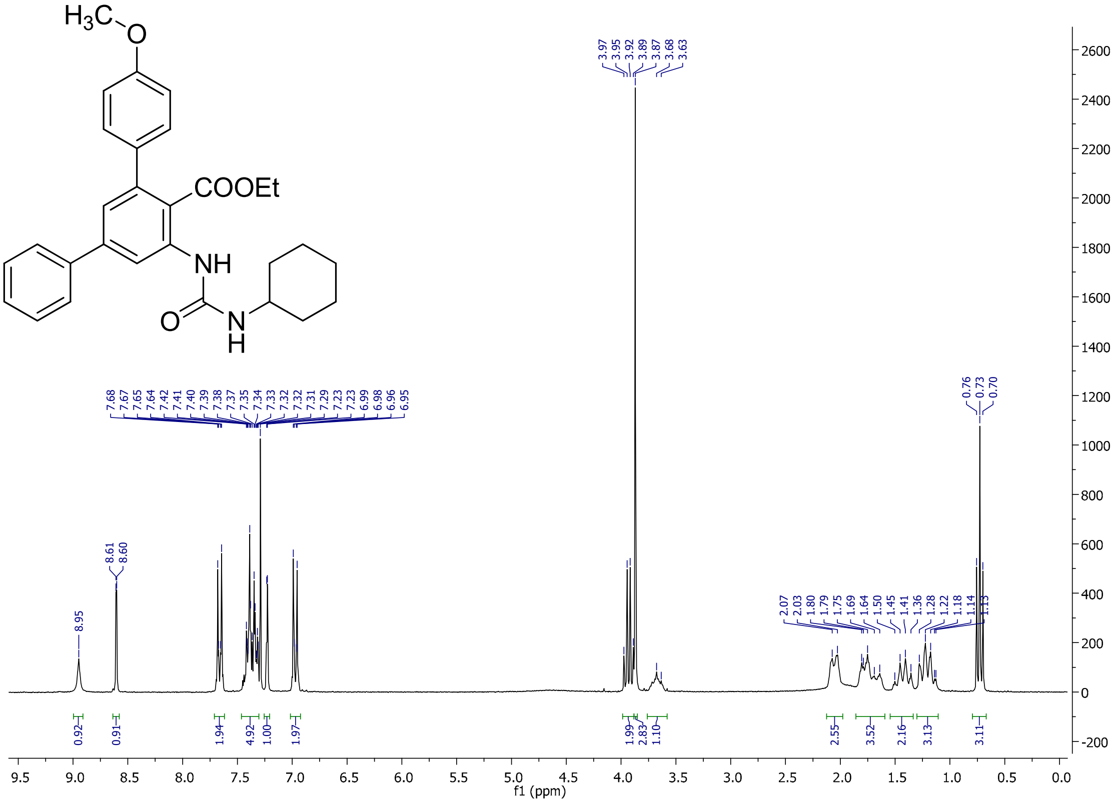

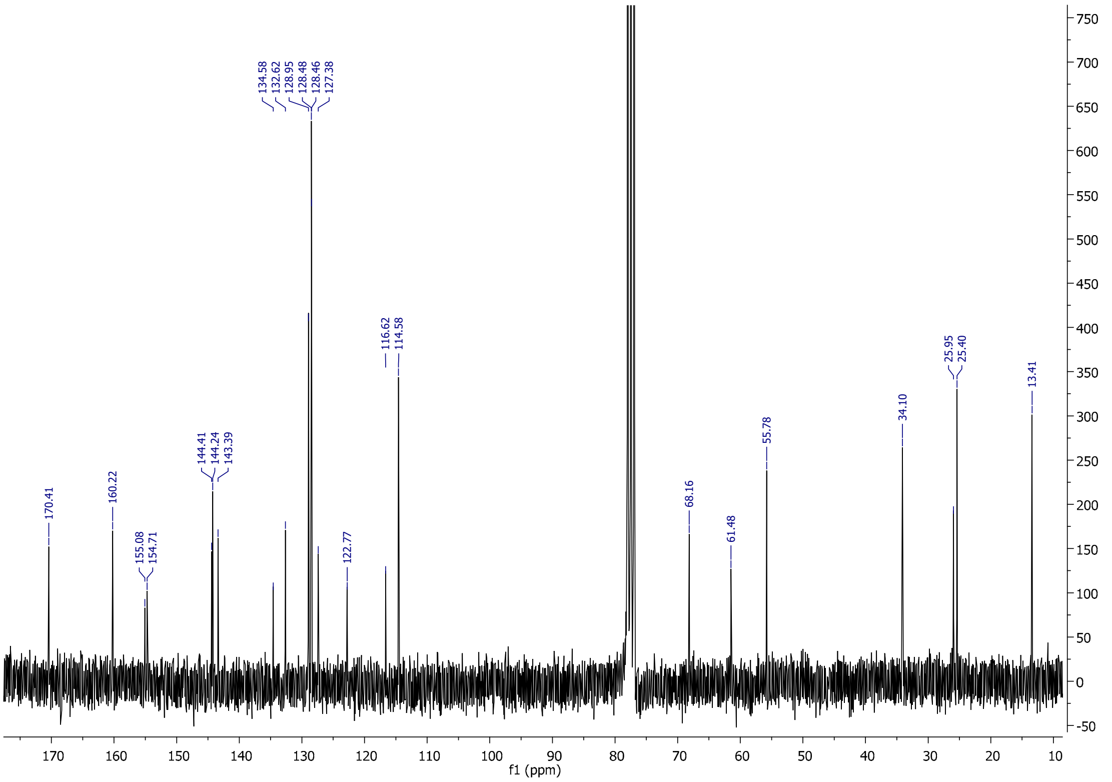

Supplement: Supplementary file 1 [file molecules-28-05374-s001.zip › molecules-2478952-supplementary.pdf]
